# A R2R3-MYB gene-based marker for the non-darkening seed coat trait in pinto and cranberry beans (*Phaseolus vulgaris* L.) derived from ‘Wit-rood boontje’

**DOI:** 10.1007/s00122-020-03571-7

**Published:** 2020-02-28

**Authors:** M. Erfatpour, K. P. Pauls

**Affiliations:** Department of Plant Agriculture, University of Guelph, Guelph, ON N1G 2W1 Canada

## Abstract

**Key message:**

The gene *Phvul.010G130600* which codes for a MYB was shown to be tightly associated with seed coat darkening in *Phaseolus vulgaris* and a single nucleotide deletion in the allele in Wit-rood disrupts a transcription activation region that likely prevents its functioning in this non-darkening genotype.

**Abstract:**

The beige and white background colors of the seed coats of conventional pinto and cranberry beans turn brown through a process known as postharvest darkening (PHD). Seed coat PHD is attributed to proanthocyanidin accumulation and its subsequent oxidation in the seed coat. The *J* gene is an uncharacterized classical genetic locus known to be responsible for PHD in common bean (*P. vulgaris*) and individuals that are homozygous for its recessive allele have a non-darkening (ND) seed coat phenotype. A previous study identified a major colorimetrically determined QTL for seed coat color on chromosome 10 that was associated with the ND trait. The objectives of this study were to identify a gene associated with seed coat postharvest darkening in common bean and understand its function in promoting seed coat darkening. Amplicon sequencing of 21 candidate genes underlying the QTL associated with the ND trait revealed a single nucleotide deletion (c.703delG) in the candidate gene *Phvul.010G130600* in non-darkening recombinant inbred lines derived from crosses between ND ‘Wit-rood boontje’ and a regular darkening pinto genotype. In silico analysis indicated that *Phvul.010G130600* encodes a protein with strong amino acid sequence identity (70%) with a R2R3-MYB-type transcription factor *Mt*PAR, which has been shown to regulate proanthocyanidin biosynthesis in *Medicago truncatula* seed coat tissue. The deletion in the ‘Wit-rood boontje’ allele of *Phvul.010G130600* likely causes a translational frame shift that disrupts the function of a transcriptional activation domain contained in the C-terminus of the R2R3-MYB. A gene-based dominant marker was developed for the dominant allele of *Phvul.010G130600* which can be used for marker-assisted selection of ND beans.

**Electronic supplementary material:**

The online version of this article (10.1007/s00122-020-03571-7) contains supplementary material, which is available to authorized users.

## Introduction

Common bean (*Phaseolus vulgaris* L.) is the most important food legume crop. It is grown worldwide for its nutrient-rich green pods or mature seeds (shell beans and dry beans). Dry beans are rich sources of vitamins and minerals, as well as plant phytochemicals. Different market classes of dry beans (including black, white navy, dark red kidney, light red kidney, pinto, cranberry, and many others) are recognized and bred that are based on the physical properties of their seeds, including seed coat color and pattern, seed size, and seed shape.

Flavonoids represent one of the largest classes of plant phenolic compounds. More than 9000 individual compounds, characterized by a 15-carbon skeleton, arranged in two aromatic rings connected by a three-carbon bridge (C6–C3–C6) have been identified (Buer et al. 2010). Flavonoids are further divided by the nature of the C3 element into major subclasses, including chalcones, flavones, flavonols, flavan 3-ols, isoflavonoids, anthocyanins, and proanthocyanidins (PAs) (Davies and Schwinn 2006).

These compounds have a variety of biological activities in humans and plants. As dietary components, flavonoids are believed to enhance human health due to their antioxidant, hepatoprotective, antiviral, antibacterial, anti-inflammatory, and anticancer activities (Mishra et al. 2013; van Dam et al. 2013; Cushnie and Lamb 2005; Pan et al. 2010; Koen et al. 2005). Flavonoids participate in a wide range of physiological activities in plants, including defense against herbivores, pests, and pathogens (Bennett and Wallsgrove 1994), UV-light protection (Cetin 2014; Zhao et al. 2017), attraction of insect pollinators and fruit-dispersing animals, signaling to rhizobial bacteria (Cipollini and Levey 1997; Wink 2008), and allelopathism (Li et al. 2010).

These phenolic secondary metabolites are synthesized as part of the larger phenylpropanoid pathway, the flavonoid biosynthesis pathway, by the activity of a multienzyme complex (flavonoid metabolon) which is weakly associated with the cytoplasmic face of the endoplasmic reticulum (ER) (Hrazdina and Wagner 1985). The early steps in the pathway begin with the condensation of three molecules of malonyl-CoA with one 4-coumaroyl-CoA molecule to yield chalcone by chalcone synthase (CHS) (Clegg and Durbin 2003). Chalcone then is converted to naringenin by chalcone isomerase (CHI) (Shimada et al. 2003). From this point on, the pathway diverges into several branches leading to the synthesis of different flavonoid compounds according to the plant species, developmental stage, and growth conditions (Debeaujon et al. 2001). Two main branches of the flavonoid pathway, leading to the production of proanthocyanidins (PAs) and anthocyanins, share two more steps, including hydroxylation of naringenin to dihydroflavonols (dihydrokaempferol, dihydroquercetin, and dihydromyricetin) by flavanone-3-hydroxylase (F3H) and flavonoid 3ʹ-hydroxylase (F3ʹH), and reduction of dihydroflavonols to leucoanthocyanidins by dihydroflavonol reductase (DFR). From this step onward, the pathway diverges into the PA branch and the anthocyanin branch.

The PAs, also called condensed tannins, are oligomers or polymers of 2,3-*cis*-flavan-ols (e.g., (−)-epiafzelechin, (−)-epicatechin, and (−)-epigallocatechin) and/or 2,3-*trans*-flavan-3-ols (e.g., (+)-afzelechin, (+)-catechin, and (+)-gallocatechin) (Davies and Schwinn 2006). The two most common monomeric units of PAs are (−)-epicatechin and (+)-catechin (He et al. 2008). In addition to DFR, PA biosynthesis is dependent upon leucoanthocyanidin reductase (LAR), anthocyanidin synthase (ANS), and anthocyanidin reductase (ANR). LAR catalyzes the conversion of leucoanthocyanidins to 2,3-*trans*-flavan-3-ols through the PA branch, and ANR catalyzes the conversion of anthocyanidins (synthesized from the conversion of leucoanthocyanidins by ANS through the anthocyanin branch) to 2,3-*cis*-flavan-ols (Davies and Schwinn 2006).

Monomer composition and the degree of polymerization of PAs, as well as the positions and stereochemistries of the carbon–carbon interflavan bonds, vary in different plant species and in different tissues and developmental stages of a single plant (Tanner et al. 2003). For example, PAs are predominantly composed of (–)-epicatechin units in the seeds of the model plants *Arabidopsis thaliana* and *Medicago truncatula* and in the leaves of the legume *Lotus corniculatus*, while PAs contain both catechin and epicatechin in *P*. *vulgaris* and *Pisum sativum* (pea) seeds (Beninger et al. 2005; Chen et al. 2015; Ferraro et al. 2014).

In seeds, PAs are synthesized in the inner integument or endothelium layer of the seed coat during development (Smýkal et al. 2014) and localize to the cell walls during maturation (Zhao et al. 2010). The presence of PA precursors, catechin and epicatechin, in the seed coat has been associated with seed coat postharvest darkening (PHD) phenomenon in pinto beans and cranberry beans (Beninger et al. 2005; Chen et al. 2015). Because a light seed coat color is considered by consumers to be an indicator of quality or freshness (Nasar-Abbas et al. 2009), darkened seeds have reduced value in the market. It is worth mentioning that flavan-3-ols and their polymers have been associated with sensory properties of bitterness and astringency (Robichaud and Noble 1990). As the degree of polymerization of flavan-3-ols increases, bitterness decreases, and astringency increases (Peleg et al. 1999).

The structural genes encoding PA biosynthetic enzymes and their regulatory factors have been well characterized in several model plant species including *Arabidopsis*, maize (*Zea mays*), snapdragon (*Antirrhinum majus*), petunia (*Petunia hybrida*), and *Medicago* (Mol et al. 1998; Quattrocchio et al. 2006; Schwinn et al. 2006; Pang et al. 2007). This information has been used to aid genetic elucidation of PA biosynthesis in other plant species. The genes underlying the *Arabidopsis* flavonoid pathway are mostly single-copy genes named *Transparent TESTA* (*TT*), making this species a unique organism to understand the major branch pathways of flavonoid biosynthesis and their subcellular localization (Winkel-Shirley 2001). However, the genetic basis of PA biosynthesis, leading to the seed coat PHD in two market classes of *P. vulgaris*, including pinto beans and cranberry beans, remains unelucidated.

PA precursors are transported from the cytosolic surface of the ER to the vacuole. At least three non-exclusive mechanisms have been suggested for the intracellular and/or extracellular transport of flavonoid compounds: (1) membrane-mediated transport, (2) vesicle trafficking, and (3) glutathione *S*-transferase (GST)-mediated transport (Zhao 2015). Two multi-drug and toxic compound extrusion (MATE) transporters, MATE and MATE1, are vacuolar membrane-localized transporters in *Arabidopsis TT12* and *Medicago*, respectively. They mediate vacuolar uptake of epicatechin 3-*O*-glucoside (glycosylated 2,3-*cis*-flavan-3-ol) for PA biosynthesis in the seed coat (Debeaujon et al. 2001; Zhao and Dixon 2009). The *Arabidopsis TT9* gene encodes a Golgi-localized vesicle trafficking factor, GFS9, which contributes to deposition of PAs in vacuoles of the seed coat (Ichino et al. 2014). The *Arabidopsis TT19* encodes a GST protein with possible involvement in vacuolar transport of PA precursors (Kitamura et al. 2004).

During aging, vacuolar and apoplastic phenolic substrates such as the flavonols (kaempferol and quercetin) and the flavan-3-ols [(+)-catechin and (−)-epicatechin] are oxidatively transformed to brown components by the activity of peroxidase (POD) and polyphenol oxidase (PPO) enzymes, as well as autoxidation (Vaughn and Duke 1984; Jiménez and García-Carmona 1999; Laukkanen et al. 1999; Takahama and Hirota 2000; Takahama 2004). *AtTT10* and eight *BnTT10* genes have been proposed to be involved in these reactions in *Arabidopsis* and three *Brassica* species (three from *B*. *napus*, three from *B*. *rap*a, and three from *B*. *oleracea*), respectively (Pourcel et al. 2005; Zhang et al. 2013).

The genes encoding the flavonoid biosynthetic enzymes are classified as early biosynthetic genes (EBGs) (*CHS*, *CHI*, and *F3H*) and late biosynthetic genes (LBGs) (*DFR*, *ANS*, *ANR*, and *LAR*) (Nesi et al. 2001; Winkel-Shirley 2001; Lepiniec et al. 2006). The expression of LBGs involved in PA biosynthesis in the seed coat is mainly regulated by a ternary MYB-bHLH-WD40 (MBW) protein complex which is formed by highly conserved transcription factors, including a MYB interacting with MYC-like basic helix-loop-helix (bHLH) proteins and WD40-repeat proteins (Baudry et al. 2004). Generally, transcription factors have modular structures and contain two functional domains, a highly conserved DNA-binding domain which binds to the promoters of the target genes, and a highly variable C-terminal region which contains binding sites for interaction with other proteins such as coregulatory proteins (Liu et al. 1999). DNA-binding domains have been widely used to classify prokaryotic and eukaryotic transcription factors into families. MYB DNA-binding domains are generally composed of 1-4 imperfect amino acid sequence repeats (R) of 50–53 amino acids (Chen et al. 2006; Jiang et al. 2004a, b; Ogata et al. 1996; Stracke et al. 2001). Based on the number of repeats found in the DNA-binding domain, MYB proteins are classified into four subfamilies, including one repeat or MYB-like proteins (R1MYB); two repeats (R2R3-MYB); three repeats (R1R2R3-MYB); and four repeats (R0R1R2R3-MYB) (Du et al. 2012; Jiang et al. 2004a, b). Each repeat contains three conserved tryptophan (W) residues spaced 18 or 19 amino acids apart, as well as three α-helices with the second and the third helices forming a helix-turn-helix structure (Ogata et al. 1996). The first tryptophan of the R3 repeat can be replaced by hydrophobic amino acids such as isoleucine (I) and phenylalanine (F) (Ambawat et al. 2013; Du et al. 2012). The third helices of the R2 and R3 repeats serve as ‘recognition helices’ that make direct contact with the major groove of the promoter DNA (Jia et al. 2004).

In *Arabidopsis*, TT2, a R2R3-MYB transcription factor, TT8, a bHLH transcription factor, and TRANSPARENT TESTA GLABRA1 (TTG1), a WD40 protein, form an MBW regulatory complex which plays the main role in production of PA in the endothelium of wild-type seeds by regulating the expression of: *DFR*, *ANS*, *BAN*, *TT19*, *TT12*, and *AHA10* (Xu et al. 2014). The MYB5–TT8–TTG1 protein complex is another MBW which regulates the expression of *DFR*, *ANS*, and *TT12* for PA accumulation in *Arabidopsis* seed coat (Xu et al. 2014).

In the seed coat of *Medicago*, a MBW complex consisting of *Mt*PAR (R2R3-MYB), *Mt*TT8 (bHLH), and *Mt*WD40-1 (WD40 protein) regulates the expression of two key structural genes, *ANR* and *LAR*, leading to PA biosynthesis (Pang et al. 2009; Verdier et al. 2012; Li et al. 2016). Moreover, a quaternary complex has been suggested for the regulation of PA biosynthesis in the seed coat of *Medicago* in which *Mt*MYB5 and *Mt*MYB14 activate the *ANR* and *LAR* genes synergistically in the presence of *Mt*TT8 and *Mt*WD40-1 proteins (Liu et al. 2014).

Three distinct phenotypes have been defined in beans based on the rapidity and intensity of seed coat PHD they undergo (Elsadr et al. 2011), namely: (1) regular darkening (RD) which darken during normal storage within a few weeks after harvest or within 24 h after exposure to UV light (Junk-Knievel et al. 2008), (2) slow darkening (SD) which require distinctly longer periods for the seed coat to exhibit PHD than RD beans, and (3) non-darkening (ND) which never darken with age. These phenotypes are determined by interactions between two loci identified by classical segregation studies, including the *J* locus which contributes to mature color development in RD seed coats (Bassett 1996a, b) and the *Sd* locus which determines how quickly a seed coat will darken in plants with a *J* genotype (Junk-Knievel et al. 2008). Plants that are homozygous recessive at *J* do not form brown pigmentation during seed maturation and after harvest (Bassett 2007; Elsadr et al. 2011; Erfatpour et al. 2018).

The STS marker OL4S_500_ was linked to *J* on chromosome Pv10 (McClean et al. 2002). Using a recombinant inbred line (RIL) population derived from a cross between a ND cranberry-like bean ‘Wit-rood boontje’ and a SD pinto bean (1533-15), Erfatpour et al. (2018) mapped a QTL associated with the ND trait in an interval flanked by single nucleotide polymorphism (SNP) markers ss715646341 (Pv10:29.74 cM) and ss715647913 (42.27 cM) on chromosome Pv10 of the genetic map and between SNPs ss715646348 (Pv10:40 164,667 bp) and ss715647917 (Pv10:40 295,580 bp) on the physical assembly. In addition to the OL4_500_ marker previously linked to *J*, forty candidate genes were identified in the ND QTL region including three genes that are encode ‘MYB proteins’ (Erfatpour et al. 2018). The *Sd* locus was mapped on chromosome Pv07 in a different study (Felicetti et al. 2012) and in the study based on the ‘Witrood boontje’ × 1533-15 RILs (Erfatpour et al. 2018) confirming that the genetic locus controlling the SD trait differs from the ND trait.

There is limited information about the biochemistry, structural genes, and regulatory genes contributing to the seed coat PHD in *P. vulgaris* seeds. The seed coat of a RD pinto bean, CDC Pintium, was found to contain higher levels of kaempferol and PAs as compared to SD 1533-15 (Beninger et al. 2005). Formation and subsequent oxidation of kaempferol-catechin dimers was the proposed mechanism for seed coat PHD in pinto beans (Beninger et al. 2005). Marles et al. (2008) reported a higher concentration of kaempferol and PPO activity in the seed coat of RD pinto beans compared to SD samples. It was found that the seed coat of ND cranberry beans do not contain catechin, epicatechin, or PAs (Chen et al. 2015). Transcript levels of flavonoid biosynthetic genes, *PvF3H1*, *PvDFR1*, *PvDFR2*, *PvLAR*, *PvANS*, *PvANR1*, *PvMATE1*, and *PvMATE2* were found to be significantly higher at early and intermediate stages of seed development in the seed coat of a RD cranberry bean compared to a ND cranberry bean and their expression levels were coordinately increased with transcript levels of *Pv*MYB6 (*Phvul.006G114800*), *Pv*MYB9 (*Phvul.011G105600*), and *Pv*MYB11 (*Phvul.003G222400*) (Freixas Coutin et al. 2017).

*PvANR1* was reported to be more strongly associated with proanthocyanidin levels in the seed coat of RD cranberry bean than *PvF3H1* and *PvDFR1*, consistent with contribution of *ANR* genes to PA accumulation in seed coats of pea, soybean, *Medicago*, *Arabidopsis*, and *B*. *napus* (Debeaujon et al. 2003; Auger et al. 2009; Pang et al. 2007; Kovinich et al. 2012; Ferraro et al. 2014; Freixas Coutin et al. 2017). Although *PvLAR* and *MtLAR* are expressed in the seed coat tissue in *P. vulgaris* and *Medicago*, respectively, the roles of these genes’ products in seed coat PA biosynthesis remains unknown (Pang et al. 2007; Freixas Coutin et al. 2017).

This research aimed to (1) determine a variant or set of variants in candidate genes previously identified in the ND QTL that might identify it as the gene regulating seed coat PHD in common bean, (2) predict the functional effect of the genetic variant(s) on PA biosynthesis, and (3) develop a gene-based marker that can be utilized in marker-assisted selection (MAS) in early stages of a breeding program for ND beans.

## Materials and methods

### Plant material

Six different genotypes including ‘Wit-rood boontje,’ 1533-15, RIL29, RIL81, Othello, and Etna, were used for target resequencing. ‘Wit-rood boontje’ is a plant introduction (PI439540) obtained from the USDA National Centre for Genetic Resources Preservation at Ft. Collins, CO, which has very pale pink, cranberry bean-like stripes on a white background (Erfatpour et al. 2018). 1533-15 is a slow darkening pinto bean variety from the Crop Development Center (CDC), of the University of Saskatchewan (Felicetti et al. 2012). RIL29 with ND seed coat phenotype and RIL81 with regular darkening seed coat phenotype are two F_2_-derived F_6_ RILs from a ‘Wit-rood boontje’ × 1533-15 cross (Erfatpour et al. 2018). Othello and Etna are regular darkening pinto bean and regular darkening cranberry bean varieties, respectively. A population of 128 F_2_-derived F_6_ RILs developed from the ‘Wit-rood boontje’ × 1533-15 cross plus 38 commercial varieties and breeding lines of pinto beans and cranberry beans were used to assess the efficiency of a SNP-based marker. The varieties and lines used for marker screening were: AC Island, Burdett, Centennial, Croissant, Jackpot, Kodiak, Kimberly, La Paz, Lariat, Marmot, ME70, ME98, ME105, ME128, ME145, ME182, ME233, Othello, P13HR088, PT11-9, Santa Fe, Sequoia, Shoshone, Stampede, UI 114, Windbreaker, and 16-NDP1 from pinto beans, and CBX 1148, C13HR118, C15HR009, C15HR185, Etna, OAC Candycane, OAC Racer, P12HR1410, Red Rider, SVMTH, and 94CTCOOP-8184 from cranberry beans. The seed coat phenotypes were determined by visual screening of UV treated seeds (Junk-Knievel et al. 2008).

### Targeted resequencing

Candidate genes in the QTL region were prioritized based on their function annotations obtained from the *P. vulgaris* v2.1 reference assembly available in Phytozome v12.1 (https://phytozome.jgi.doe.gov). Targeted resequencing of the most promising genes for the seed coat PHD trait was performed using Illumina Next Generation Sequencing (NGS) with MiSeq system. The instructions for Illumina targeted resequencing, including amplicon generation, library preparation, amplicon sequencing, and data analysis (www.illumina.com/technology/next-generation-sequencing.html) were followed as described at the Illumina website (http://www.illumina.com).

### Amplicon generation

DNA samples from the genotypes were isolated using CTAB extraction protocol (Doyle and Doyle 1990) and quantified using a Qubit fluorometer. The Integrated DNA Technologies PrimerQuest Tool (https://www.idtdna.com/PrimerQuest/Home/Index) was used to design primers. Amplicons were generated from the promising genes in each genotype using polymerase chain reactions (PCRs) in volumes of 20 μl containing: 10 ng template DNA, 1 × PCR buffer, 1.5 mM magnesium chloride (MgCl_2_), 0.2 mM dNTPs, 0.5 µM forward and 0.5 µM reverse primers, and 1 U of Taq polymerase. The PCRs were performed in a Bio-Rad Laboratories, Inc. (USA) thermal cycler with a program that included: an initial denaturation step for 2 min at 94 °C, followed by 35 cycles each of 1 min at 94 °C (denaturation), 1 min at annealing temperature of primer (annealing), 1 min at 72 °C (extension), and a final extension of 7 min at 72 °C. The amplicons were electrophoretically separated with 0.8% agarose gels at 90 V for 2 h, stained with ethidium bromide (0.5 µl/ml of agarose gel), and visualized with UV transillumination. The DNA bands with expected sizes were cut from the agarose gels, purified using Zymoclean™ DNA Gel Recovery Kit (ZYMO RESEARCH), quantified by Qubit and normalized to 2.5 ng/µl.

### Library preparation

Amplicons from each genotype were pooled and 50 ng of DNA (25 ul of 2.5 ng/ul) from each pool were used for library preparation, using the Illumina Nextera^®^ DNA Library Prep Kit as described in the reference guide (Document # 15027987 v01). The library prep workflow included a ‘tagmentation’ reaction which simultaneously fragmented and tagged the amplicons with adapter sequences using the Nextera transposome. The tagmented fragments were purified from the Nextera transposome and PCR amplified to add Index 1 (i7) and Index 2 (i5) adapters required for cluster generation and sequencing to each fragment. AMPure XP beads were used to purify the libraries for each genotype and remove short library fragments. The size distributions of the fragments in the amplicon libraries were determined with an Agilent Technology 2100 Bioanalyzer. The optimal fragment sizes range from ~ 250  to 1000 bp. The libraries were normalized to 4 nM, and equal volumes were pooled.

### Amplicon sequencing

The prepared library was denatured and diluted to a final volume of 600 ul using MiSeq Reagent kit v3, 600 cycles (Catalog # MS-102-3003) and following the MiSeq System Denature and Dilute Libraries Guide (Document # 15039740 v01). A PhiX library was prepared, denatured, and diluted for use as a sequencing control. The Library (594 ul) and PhiX control (6 ul) were combined and loaded onto the prefilled reagent cartridge according to the Reagent Preparation Guide (Part # 15044983 Rev.). The MiSeq instrument was set up according to the MiSeq System Guide (Document # 15027617 v03 Material # 20024228) and paired-end 300-cycle per read sequencing was performed.

## Data analysis

FASTQC software (Andrews 2010) was used to control the quality of the Illumina output and sequences were aligned to the *P. vulgaris* reference genome using CLC Genomics Workbench 11 (http://www.qiagenbioinformatics.com). Multiple DNA and protein sequence alignments were performed with Clustal Omega (http://www.ebi.ac.uk/Tools/msa/clustalo/). Protein–protein BLAST (blastp) analysis was performed in NCBI to query the non-redundant protein sequence (nr) database (https://blast.ncbi.nlm.nih.gov/Blast.cgi). A phylogenetic tree was built using the neighbor-joining algorithm with Mega7.0 software (http://www.megasoftware.net/).

## Results

Twenty-one candidate genes were selected out of the genes that are annotated in the QTL region identified in previous work to be associated with the PHD trait (Erfatpour et al. 2018) as the most likely genes to be control this trait. The information regarding the annotation of the selected genes and the primer sets used for amplicon generation is shown in Table 1 and supplementary Table S1, respectively.

**Table 1:** Twenty-one candidate genes prioritized for sequencing

Gene ID	Annotation	Gene size (bp)	Number of amplicon(s) per gene	Length of genomic region (bp) covered by amplicon(s)
Phvul.010G130300	Ubiquitin-like ATG12 (ATG12)	2465	2	2380
Phvul.010G130500	MYB-like DNA-binding protein	1279	1	1314
Phvul.010G130600	MYB-like DNA-binding protein	1506	1	1453
Phvul.010G130700	Transcription initiation factor TFIID Subunit 12B	9823	8	11670
Phvul.010G131300	Uncharacterized protein	5563	4	5539
Phvul.010G131400	MYB Family transcription factor TRFL6-related	6607	5	6697
Phvul.010G133100	Developmentally regulated GTP-binding protein 2	684	1	712
Phvul.010G132300	Ring/U-box domain-containing protein 2	590	1	720
Phvul.010G132500	MDC16-related	1708	1	1544
Phvul.010G132600	Apoptosis inhibitor 5-related	8359	6	7653
Phvul.010G132700	Nonsense-mediated mRNA decay protein 3	2390	1	1564
Phvul.010G132800	Protein RCC2	4719	2	2351
Phvul.010G132900	Diglyceride acyltransferase	5781	4	4674
Phvul.010G133300	Nuclear transcription factor Y subunit A-3-related	4567	4	5170
Phvul.010G133400	PHD finger protein alfin-like 6-related	4027	2	3621
Phvul.010G133500	nitrogen metabolic regulation protein NMR-related	2999	2	2756
Phvul.010G133600	Solute carrier family 25	2370	1	1158
Phvul.010G133700	Domain of unknown function	3531	2	26,40
Phvul.010G133800	Domain of unknown function	1257	1	1154
Phvul.010G133900	Domain of unknown function	1323	1	1260
Phvul.010G134000	Domain of unknown function	552	1	1043
Total			51	67,073

The table includes their gene name (Phytozome v12.1), annotations according to *P*. *vulgaris* v2.1, sizes (bp), the number of PCR amplicons generated for each gene, and the length of genomic region (bp) covered by amplicon(s)

The *Phvul.010G130300* gene was annotated as ‘autophagy-related gene 12 (*ATG12*)’ which is known for its roles in plant autophagic nutrient recycling during senescence and survival under N- and C-limiting conditions (Doelling et al. 2002; Thompson et al. 2005) through its attachment to *ATG5* by a ubiquitin-like conjugation system. Plants with enhanced autophagy show elevated levels of expression of genes involved in anthocyanin and flavonoid biosynthesis (Masclaux-Daubresse et al. 2014; Minina et al. 2018). The genes *Phvul.010G130500*, *Phvul.010G130600*, and *Phvul.010G131400* were annotated as ‘MYB proteins’ which are involved in transcriptional regulation of PA biosynthesis in a variety of plant species (Akagi et al. 2009; Koyama et al. 2014; Lu et al. 2017) and were the most interesting candidates in the present study. *Phvul.010G130700* and *Phvul.010G133300* were annotated as ‘transcription initiation factor (TFIID) subunit 12B’ and ‘nuclear transcription factor Y subunit A-3-related,’ respectively. Both genes are general (basal) transcription factors which play key roles in the regulation gene expression of a subset of genes (Robles et al. 2007; Kabe et al. 2005). *Phvul.010G131300*, *Phvul.010G133700*, *Phvul.010G133800*, *Phvul.010G133900*, and *Phvul.010G134000* are genes of unknown function. The *Phvul.010G133100* gene was annotated as a ‘developmentally regulated GTP-binding protein 2 (DRG2)’ (formerly known as DRG1) which is a class of signal-transducing GTPases with possible roles in vesicle transport (Assmann 2002) and heat stress responses (Stafstrom 2008). The *Phvul.010G132300* gene possess ‘E3 ubiquitin ligase activity’ which can participate in a range of functions, from self-incompatibility (Stone et al. 2003) and hormone responses (Amador et al. 2001; Samuel et al. 2008) to biotic (Heise et al. 2002; Kirsch et al. 2001) and abiotic stress responses (Cho et al. 2006; Samuel et al. 2006). The proteins encoded by the *Phvul.010G132500* and *Phvul.010G132600* genes are uncharacterized. The *Phvul.010G132700* gene was annotated as a ‘Nonsense-mediated mRNA decay (NMD) protein’ that controls the quality of mRNA via regulation of gene expression and destruction of aberrant mRNA and may function in plant pathogen responses (Rayson et al. 2012). *Phvul.010G132800* was annotated as a ‘regulator of chromosome condensation 2 (RCC2),’ which plays a functional role in cells undergoing mitosis (Yenjerla et al. 2013). The *Phvl.010G132900* gene was annotated as a ‘diglyceride acyltransferase (DGAT)’ which catalyzes the synthesis of triacylglycerol (Ohlrogge and Browse 1995). *Phvul.010G133400* was annotated as a ‘PHD finger protein,’ which participates in transcriptional regulation activity (Wilson et al. 2001), chromatin regulation (Aasland et al. 1995) and confers abiotic stress tolerance in *Arabidopsis* (Wei et al. 2015). *Phvul.010G133500* encodes a nitrogen metabolic regulation protein which may influence the C/N ratio and can be correlated with expression of the late flavonoid/anthocyanin biosynthetic genes, *ANS*, *UFGT* and *FLS* (Wan et al. 2015). *Phvul.010G133600* was annotated as a ‘solute carrier family 25 (SLC25)’ gene which is a mitochondrial transporter that regulates the transport of many types of substances into or out of mitochondria (Höglund et al. 2011).

**Fig. 1:**
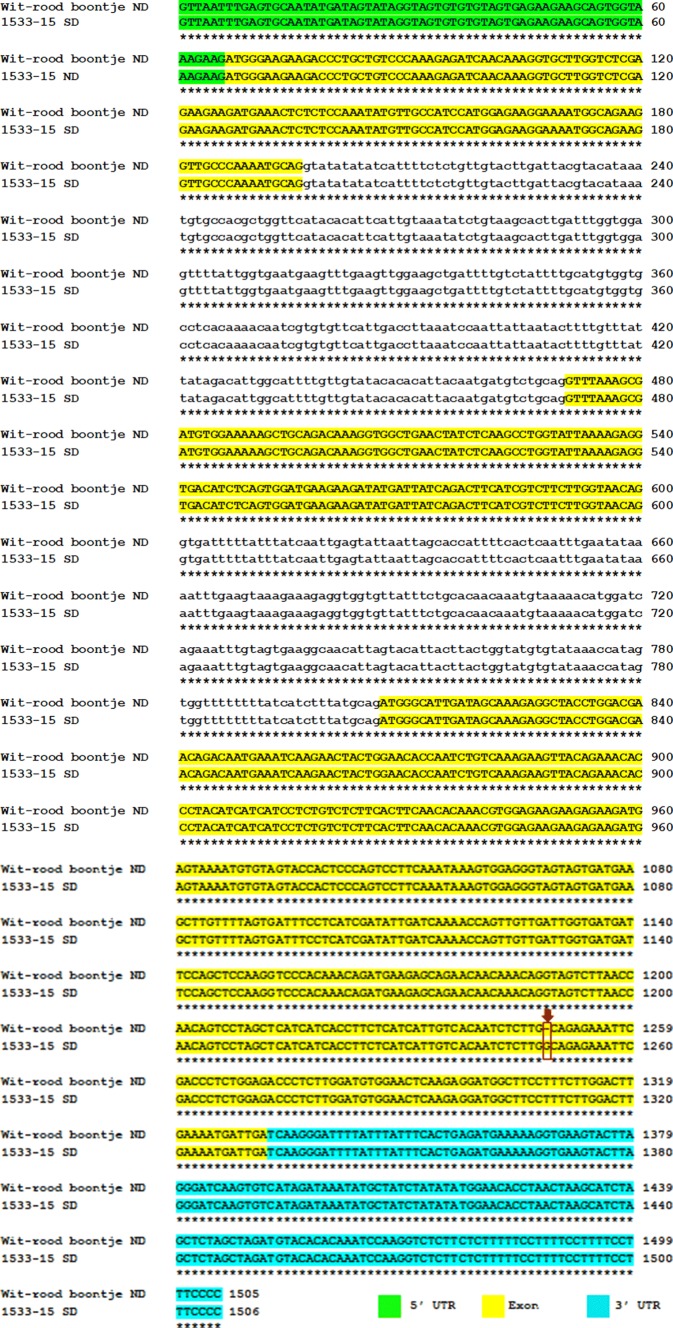
Sequence alignment of the gene *Phvul.010G130600* in ‘Wit-rood boontje’ [parent with the non-darkening (ND) seed coat phenotype] with 1533-15 [parent with the slow darkening (SD) seed coat phenotype]. Wit-rood boontje displayed a single nucleotide deletion (c.703delG, in red box) in the 3rd exon region (yellow shaded). The green-shaded segment identifies the 5ʹ untranslated region (UTR), the blue-shaded segment identifies the 3ʹ UTR, and the lowercase letters identify the introns (color figure online)

**Fig. 2:**
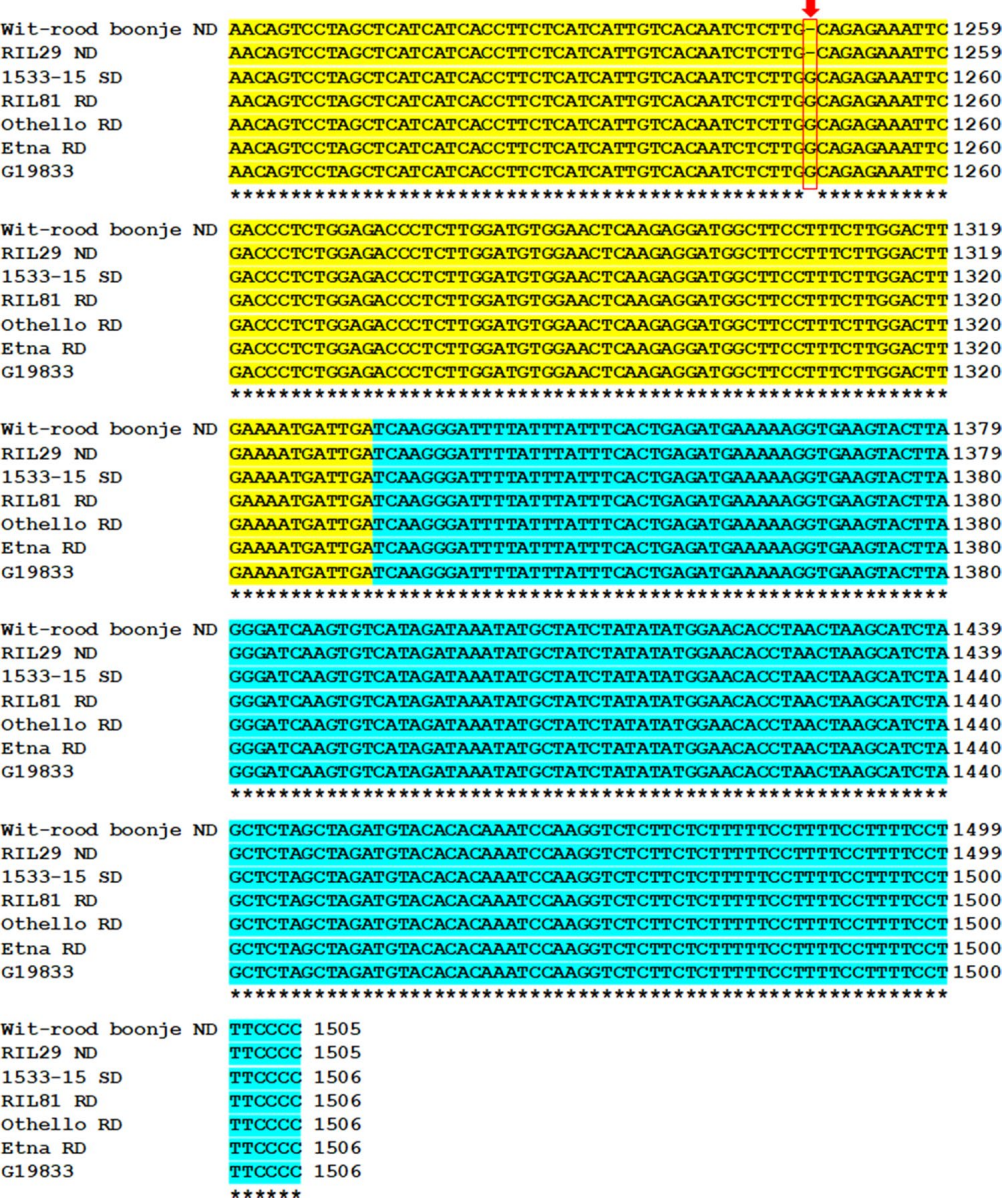
Sequence alignments of genomic segments six different genotypes corresponding to the exon region (yellow shaded) of gene *Phvul.010G130600* containing the single nucleotide polymorphism (c.703delG, bordered by red lines) associated with the non-darkening trait in ‘Wit-rood boontje.’ The sequence in Wit-rood boontje [parent A with the non-darkening (ND) seed coat phenotype] is compared to: RIL29 (ND), 1533-15 [parent B with the slow darkening (SD) seed coat phenotype], RIL81 [regular darkening (RD)], Othello (RD pinto bean variety), Etna (RD cranberry variety), and G19833 (*P*. *vulgaris* reference genome G19833) (color figure online)

In total, 51 PCR amplicons were successfully generated and sequenced, covering 67,073 bp of the QTL region and representing 21 candidate genes (Table 1). The FASTQC analysis indicated that the per base sequence quality score was high (> Q30) and the mean quality score per read was over 36 (Figure S1), both results indicating that the raw sequencing data were of good quality. DNA sequence analysis revealed single and multiple nucleotide polymorphisms distributed either within coding sequences (exons) or non-coding sequences (introns) of most of the genes across the six different genotypes, including non-darkening ‘Wit-rood boontje’ and RIL29; slow darkening 1533-15; and regular darkening RIL81, Othello, and Etna. The results showed that for most of the detected polymorphisms in the amplicons from the candidate genes across the genotypes there was no consistent association between the genetic variants and the seed coat PHD trait (Figure S2). However, there was a single nucleotide polymorphism that was located in the coding region of gene *Phvul.010130600* that did differentiate the individuals with the ND seed coat phenotype from the ones with SD and RD seed coat phenotypes. The polymorphism represents a deletion of a guanine from the sequence of ‘Wit-rood boontje’ and RIL29 (which is ND) in c.703delG (Figs. 1, 2). *Phvul.010130600* is annotated as ‘MYB transcription factor’ in the *P. vulgaris* v2.1 reference assembly (Phytozome V12.1).

**Fig. 3:**
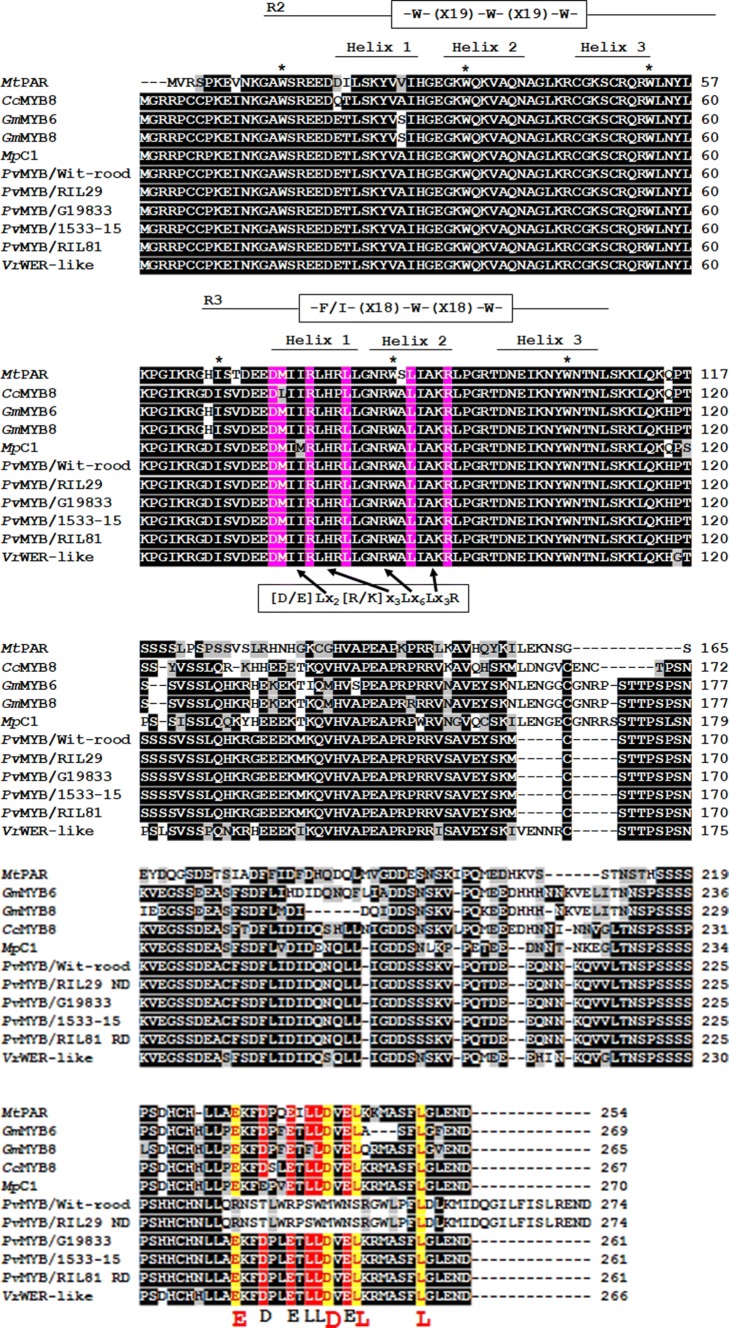
Multiple sequence alignment of R2R3-MYB proteins and putative R2R3-MYB proteins encoded by the gene homologs of *Phvul.010G130600* in *P*. *vulgaris* G19833 showing characteristic motifs. The alignment includes putative translations of homologs of *Phvul.010G130600*: in ‘Wit-rood-boontje’ [parent A with the non-darkening (ND) seed coat phenotype], 1533-15 [parent B with slow darkening (SD) seed coat phenotype], RIL81 [regular darkening (RD) line], RIL29 (ND line), *Mt*PAR (*Medtr8G020490* in *M. truncatula*), *Gm*MYB6 (*Glyma.16G007100* in *Glycine max*), *Gm*MYB8 (*Glyma.07G037700* in *Glycine max*), *Cc*MYB8 (*LOC109802394* in *Cajanus cajan*), *Mp*C1 (*C1* in *Mucuna pruriens*), and *Vr*WER-Like (*LOC106773092* in *Vigna radiata*). Motifs characteristic of MYB are identified, including the primary structure R2 and R3 DNA-binding domains [-W-(X19)-W-(X19)-W-] and [-F/I- (X18)-W-(X18)-W-]; in which W represents tryptophan and X represents any amino acid. [D/E]Lx_2_[R/K]x_3_Lx_6_Lx_3_R is the bHLH-interaction motif located on helices 1 and 2 of the R3 repeat; The EDLL motif and amino acid residues in the transactivation domain are shown, containing characteristic E (glutamic acid), D (aspartic acid), and L (leucine) amino acids

As shown in Fig. 3, the gene *Phvul.010G130600* of the reference genome *P. vulgaris* G19833 (Schmutz et al. 2014) encodes a R2R3-MYB protein with conserved DNA-binding domains R2 [-W-(X19)-W-(X19)-W-] and R3 [-F/I- (X18)-W-(X18)-W-], as well as the amino acid motif [D/E]Lx_2_[R/K]x_3_Lx_6_Lx_3_R (Phytozome V12.1). The 3 tryptophan (W) residues of the R2 repeat are at positions 16, 36, and 56, and in the R3 repeat an isoleucine residue replaces the first tryptophan (W) at position 69 and 2 additional tryptophan residues are at positions 88 and 107, respectively. The amino acid motif [D/E]Lx_2_[R/K]x_3_Lx_6_Lx_3_R is conserved among MYB proteins and located on helices 1 and 2 of the R3 repeat that interact with R/B-like bHLH factors (Zimmermann et al. 2004). The sequence for gene *Phvul.010G130600* in 1533-15 was determined to be identical to the sequence in the reference genome (data not shown) and thus the protein would have an identical amino acid sequence to that of the reference genome.

**Fig. 4:**
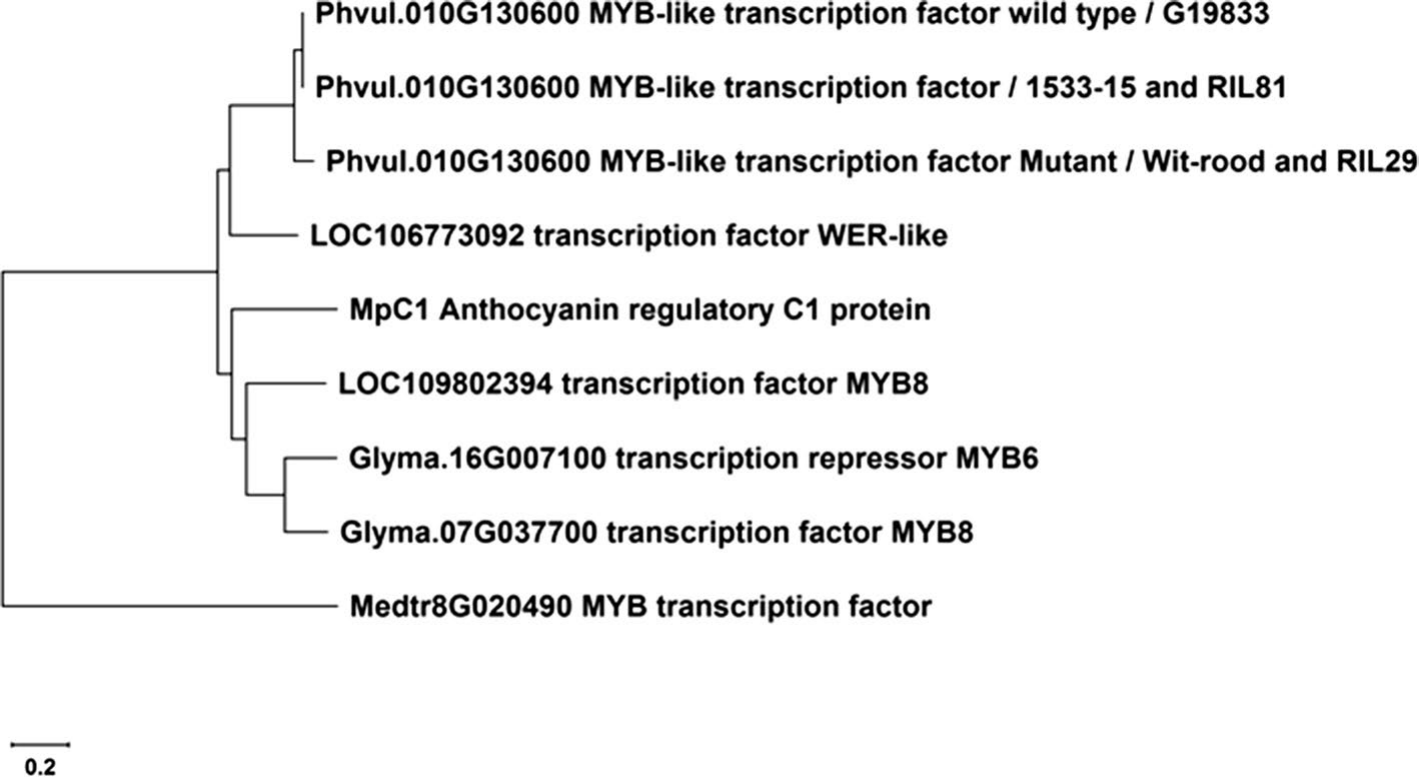
Neighbor-joining cluster analysis of putative R2R3-MYB proteins encoded by gene homologs of *Phvul.010G130600* in *P*. *vulgaris* G19833. The cluster includes the Phvul.010G130600 protein sequence: in 1533-15 [parent B with the slow darkening (SD) seed coat phenotype] and RIL81 (SD line), the Phvul.010G130600 protein sequence: in ‘Wit-rood-boontje’ [parent A with the non-darkening (ND) seed coat phenotype] and RIL29 (ND line), LOC106773092 transcription factor WER-like in *Vigna radiata*, MpC1 anthocyanin regulatory C1 protein in *Mucuna pruriens*, LOC109802394 transcription factor MYB8 in *Cajanus cajan*, Glyma.16G007100 transcription repressor MYB6 isoform X1 and Glyma.07G037700 transcription factor MYB8 in *Glycine max*, and Medtr8G020490 MYB transcription factor *Mt*PAR in *M. truncatula*

According to a protein BLAST search, the MYB encoded by the *Phvul.010G130600* exhibited 91% sequence identity with a transcription factor WER-like (*LOC106773092*) in *Vigna radiata* (mung bean) across its entire query length, followed by 83%, 82%, and 82% sequence identity with transcription factor MYB8 (*LOC109802394*) in *Cajanus cajan* (pigeon pea), an anthocyanin regulatory C1 protein (*C1*) in *Mucuna pruriens*, and a transcription repressor MYB6 isoform X1 (*Glyma.16G007100*) in *Glycine max*, respectively. It also showed 80% identity with a transcription factor MYB8 (*Glyma.07G037700*) in *G. max* and 70% identity with a MYB transcription factor *Mt*PAR (*Medtr8G20490*) in *M. truncatula* (Figs. 3, 4, Table 2).

**Table 2:** Putative homologs for Phvul.010G130600 MYB-like transcription factor in different species

Gene ID	Species	Description	Query cover (%)	Identity (%)
*LOC106773092*	*Vigna radiata*	Transcription factor WER-like	100	91
*LOC109802394*	*Cajanus cajan*	Transcription factor MYB8	100	83
*C1*	*Mucuna pruriens*	Anthocyanin regulatory C1 protein	100	82
*Glyma.16G007100*	*G. max*	Transcription repressor MYB6 isoform X1	100	82
*Glyma.07G037700*	*G. max*	Transcription factor MYB8	100	80
*Medtr8G020490*	*M. truncatula*	Transcription factor MYB	97	70

The table includes their annotations, BLAST  %query coverage, and BLAST  % identity

**Fig. 5:**
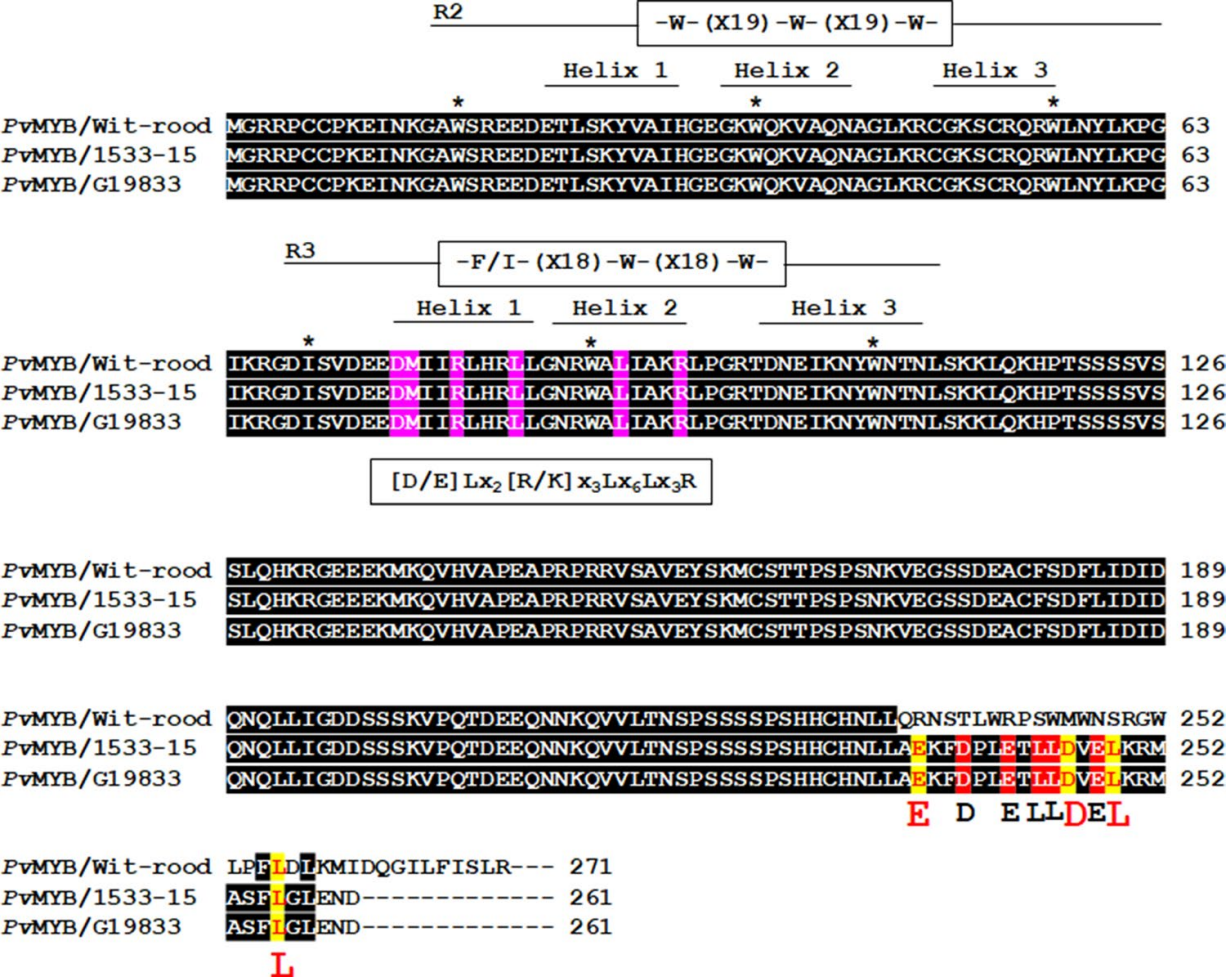
Sequence alignment of an R2R3-MYB protein encoded by the gene *Phvul.010G130600* of the reference genome *P. vulgaris* G19833 reference genome, ‘Wit-rood-boontje’ (parent A with the non-darkening (ND) seed coat phenotype), and 1533-15 (parent B with slow darkening (SD) seed coat phenotype). [-W-(X19)-W-(X19)-W-] and [-F/I- (X18)-W-(X18)-W-] are the primary structure of R2 and R3 DNA-binding domains, in which W represents tryptophan and X represents any amino acid. [D/E]Lx_2_[R/K]x_3_Lx_6_Lx_3_R is the bHLH-interaction motif located on helices 1 and 2 of the R3 repeat. E (glutamic acid), D (aspartic acid), and L (leucine) amino acids in the transactivation domain represent the EDLL motif and negatively charged residues

The SNP in the sequence for gene *Phvul.010G130600* in ‘Wit-rood boontje’ is predicted to cause a frame shift change, with alanin235 as the first affected amino acid and the new reading frame remaining open for 36 amino acids (*A235fsX*271). Therefore, the Phvul.010G130600 protein in ‘Wit-rood boontje’ would be expected to have identical DNA-binding domains to the wild-type protein, but the amino acid residues 236 to 256, which show enrichment for negatively charged residues glutamic acid (E) and aspartic acid (D) and hydrophobic residues leucine (L), in the wild-type Phvul.010G130600 protein, will be disrupted in the protein encoded by the ND allele because of the frame shift caused by the SNP (Fig. 5). In addition, the protein in the ND lines will have a longer C-terminal region (Fig. 5).

**Fig. 6:**
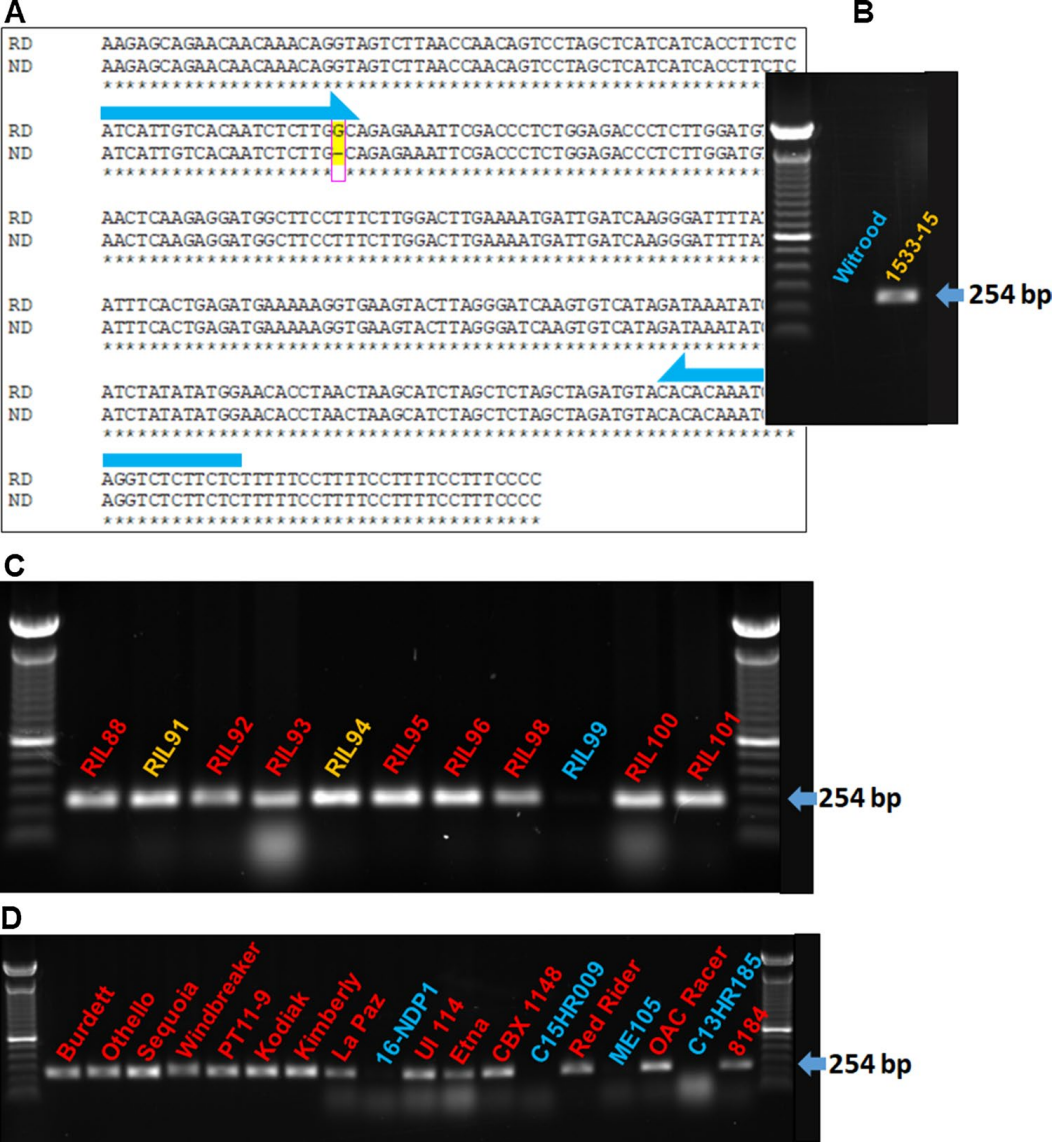
*Phvul.010G130600* SNP-based marker and the marker screen of regular, slow (SD) and non-darkening (ND) recombinant inbred lines (RIL), breeding lines and varieties of bean. **a** PCR binding sites for PCR primers (Table 3) in regular darkening (RD) and non-darkening (ND) genotypes. The SNP is identified with a red box. **b** PCRs with genomic DNA from a ND genotype like ‘Wit-rood boontje’ did not produce an amplicon. PCRs with genomic DNA from a slow darkening (SD) parent 1533-15 produced a single DNA fragment with a size of 254 bp. **c** RILs88-101 are recombinant inbred lines from a ND ‘Wit-rood-boontje’ x SD 1533-15 cross. **d** Burdett, Othello, Windbreaker, Sequoia, PT11-9, Kodiak, Kimberly, La Paz, 16-NDP1, UI 114, and ME105 are Pinto bean varieties and lines. Etna, CBX 1148, C15HR009, Red Rider, OAC Racer, C13HR185, and 8184 (94CTCOOP-8184) are cranberry bean varieties and lines. Labels that are highlighted red, yellow, and blue represent seeds with (regular darkening) RD, SD, and ND phenotypes, respectively (color figure online)

A sequence-tagged site (STS) marker was developed for the wild-type allele of the *Phvul.010G130600* gene. The primer sequences were designed to bind a site that ends with the G at c.703 that is missing in the ND genotype (Fig. 6, Table 3). A single DNA fragment with a size of 254 bp was amplified from genomic DNA from RD and SD genotypes, but not the ND genotypes (Fig. 6).

**Table 3:** PCR primer sequences for STS marker associated with the gene for the ND seed coat phenotype in common bean based on *Phvul.010G130600*

STS marker	Primer Sequence (5ʹ-3ʹ)	Linkage distance (cM) from *J* locus	Tm (°C)
PvJ-254	Forward: TCATCATTGTCACAATCTCTTGGC	0.0	63
	Reverse: GAGAAGAGACCTTGGATTTGTGTG		63

*Tm* DNA melting temperature

The occurrence of the marker was determined for the parents, ‘Wit-rood boontje’ and 1533-15, and a mapping population from a ‘Wit-rood boontje’ × 1533-15 cross, including 46 RD RILs, 31 SD RILs, and 51 ND RILs. The marker was also tested with 38 commercial varieties and breeding lines of pinto beans and cranberry beans with RD and ND seed coat phenotypes. No recombination event was observed for the marker among the test individuals; indicating that the polymorphism on which it is based is very close to or in the gene responsible for the ND trait in common bean.

## Discussion

The current results suggest that the gene *Phvul.010G130600*, which encodes a R2R3-MYB transcription factor located on chromosome Pv10, close to the STS marker OL4S_500_ which was previously found to be linked to *J* (McClean et al. 2002) and is located in the major QTL associated with the ND trait (Erfatpour et al. 2018), is responsible for the phenotypes conditioned by the *J* gene. Bassett 1996a, b described the effects of the recessive allele of the joker (*j*) gene, which include a decrease in the intensity of the background seed coat color it is introduced into and a reduction in seed coat after-darkening. The after-darkening observed in pinto and cranberry beans carrying the *J* allele has been associated with the accumulation of proanthocyanidin precursors (Beninger et al. 2005; Chen et al. 2015; Freixas Coutin et al. 2017). The after-darkening phenotype and its association with PA levels is consistent with the finding that the *Phvul010G130600* gene encodes a protein with high sequence identity to the R2R3-MYB transcription factor encoded by *MtPAR,* which plays a significant role in PA biosynthesis in *Medicago* seed coat tissue (Pang et al. 2009; Verdier et al. 2012; Li et al. 2016).

Biosynthesis of PAs involves transcriptional regulation of late biosynthetic genes in the flavonoid pathway. Among the major groups of plant transcription factors, the R2R3-MYB proteins from the MYB family play important roles in PA synthesis in the seed coat. R2R3-MYBs promote transcription of genes encoding PA synthesis enzymes when they interact with bHLH and WD40 proteins and form ternary protein complexes (Xu et al. 2015). *MtPAR* regulates ANR and LAR expression during seed development and mutants exhibit large reductions in the PA content in their mature seeds with no change in anthocyanin levels compared to the wild type, indicating that this MYB factor is specifically involved in regulating PA biosynthesis (Verdier et al. 2012). *MtPAR* is a homolog of *AtTT2*, the key regulator of PA biosynthesis in *Arabidopsis* seeds (Liu et al. 2014). Pang et al. (2007) reported that the highest level of *MtANR* expression occurred in *Medicago* seed coats, and that lower levels of expression were found in flowers and immature pods, and no expression occurred in other tissues. However, low levels of *MtLAR* expression were detected in *Medicago* flowers, pods, and seed coats (Pang et al. 2007).

Gene transcription is mediated by the enzyme RNA polymerase, which is recruited to a promoter region by general transcription factors. Its activity is modified by interactions with a multi-protein complex that includes transcriptional activators that bind DNA through DNA-binding domains and interact with the transcription complex through transcriptional activation domains (Ptashne 1988). Therefore, the specific amino acid sequence of the transcriptional activator plays a crucial role in its functionality and mutations in critical interaction domains may render it incapable of functioning in the transcription initiation complex (Figure S3). Amino acid modifications in the C-terminal regions of transcriptional activators that change the net charge of this protein region have been linked to changes in their activation rates. Estruch et al. (1994) reported that a two-amino-acid substitution in the transcriptional activation domain of the P14 peptide changed the net charge from +4 to +2 and led to 38% reduction in its activity. Alternatively, a plant protein ethylene-responsive element binding factor (ERF2) with a modified transcriptional activation domain displayed enhanced activation, when fused to DNA-binding proteins, compared to the unmodified ERF2 sequence (Li et al. 2013).

Multiple transcriptional activation domains have been identified on the basis of their amino acid compositions, including negatively charged activation domains which are rich in D and E residues and represented by the GAL4, GCN4 regulatory proteins in yeast and the herpes simplex virus VP16 protein (Lowe et al. 1985; Sadowski et al. 1988); a glutamine-rich activation domain which contains multiple repetitions such as ‘QQQXXXQQQ’ and is present in the mammalian factor SP1 (Courey et al. 1989); proline-rich activation domains which are composed of repetitions such as ‘PPPXXXPPP’ found in c-jun, AP2 and Oct-2 transcription factors of mammals (Mermod et al. 1989); an isoleucine-rich activation domain with repetitions ‘IIXXII,’ present in *Drosophila* transcription factor NTF-1 (Attardi and Tjian 1993); and metal-binding cysteine-rich activation domain present in adenovirus E1a protein (Martin et al. 1990).

Transcriptional activation and repression domains play key roles in a wide variety of biological functions by positively and negatively regulating genes involved in developmental and physiological processes in various plant species. Sainz et al. (1997), through extensive mutagenesis studies of ZmMYBC1, found that two amino acid residues, L 253 and E 262, in the C-terminus of this protein are necessary for transcriptional activity. *ZmMYBC1* encodes a R2R3-MYB protein that interacts with a bHLH protein (ZmR) to regulate anthocyanin synthesis in maize aleurone tissue (Cone et al. 1986; Goff et al. 1992; Paz-Ares et al. 1987; Rabinowicz et al. 1999). The C1-encoded protein had previously been shown to contain a negatively charged transcriptional activation domain in its C-terminal (Paz-Ares et al. 1987) which is important for the C1 function (Goff et al. 1991; Paz-Ares et al. 1990). Similarly, Docimo et al. (2016) proposed that regulatory function of *Solanum melongena MYB1* in anthocyanin biosynthesis can be impaired by a deletion of the C-terminal region. Albert et al. (2015) reported that the negatively charged C-terminal domains of the R2R3-MYB proteins involved in anthocyanin leaf markings in *Trifolium repens* are necessary to activate transcription. Members of class II ERFs such as AtERF3, AtERF4, and AtERF7 to AtERF12 in *Arabidopsis*, NtERF3 in tobacco (*Nicotiana tabacum*), and GmERF4 in soybean were identified as transcriptional repressors with ERF-associated amphiphilic repression (EAR) motifs in their C-terminal region for active repression (Fujimoto et al. 2000; Ohta et al. 2001; McGrath et al. 2005; Song et al. 2005; Yang et al. 2005; Zhang et al. 2010). EAR motif contains two distinct conservation patterns: LxLxL and DLNxxP (Kagale et al. 2010). The R2R3-MYB, MYB27, was found to be an anthocyanin repressor that functions as part of the MBW complex and represses transcription through its EAR motif (Albert et al. 2014).

Recently, ‘EDLL’ motifs, comprised of negatively charged residues, aspartic acid (D) and glutamic acid (D) and hydrophobic leucine (L), have been identified as short but strong transactivation motifs in the members of group IX of the APETALA2/ethylene response (AP2/ERF) transcription factors including *Arabidopsis* Ethylene Response Factor 98 (*AtERF98*) (Tiwari et al. 2012) and *AtERF96* (Chen et al. 2018). It has been suggested that the EDLL motif plays an important role for *AtERF96* to interact with MEDIATOR25, a subunit of the eukaryotic Mediator complex (Çevik et al. 2012), that functions as a transcriptional coactivator in *Arabidopsis* (Chen et al. 2018).

Aspartic acid and glutamic acid are among the most frequently occurring amino acids in wild‐type transactivation domains (Ravarani et al. 2018). It is possible that the EDLL motif in the C-terminal region of the R2R3-MYB encoded by *Phvul.010G130600* functions as a transactivating domain that is required to activate transcription of genes that encode enzymes in the biosynthetic pathway of proanthocyanidin synthesis in *P*. *vulgaris* seed coat.

Among the genes that exhibit high levels of protein sequence identity with the *Phvul010G130600* gene, the gene *MtPAR* is of particular interest because it has been shown to affect PA biosynthesis. *Mt*PAR is a R2R3-MYB transcription factor that plays a significant role in PA biosynthesis in *Medicago* seed coat tissue by regulating *ANR* and *LAR* expression during seed development (Pang et al. 2009; Verdier et al. 2012; Li et al. 2016). *Mtpar* mutants exhibited large reductions in the PA content of their mature seeds with no change in anthocyanin levels compared to the wild type, indicating that this MYB factor is specifically involved in regulating PA biosynthesis (Verdier et al. 2012). Pang et al. 2007 reported that the highest level of *MtANR* expression occurred in *Medicago* seed coats, and that lower levels of expression were found in flowers and immature pods, and no expression occurred in other tissues. However, low levels of *MtLAR* expression were detected in *Medicago* flowers, pods, and seed coats (Pang et al. 2007). *MtPAR* is a homolog of *AtTT2*, the key regulator of PA biosynthesis in *Arabidopsis* seeds (Liu et al. 2014).

We suggest that the MYB encoded by *Phvul.010G130600* is a PA activator that functions as part of the MBW complex and regulates transcription through its C-terminal EDLL motif. Further, we suggest that the MBW complex targets the seed coat PA biosynthesis genes encoding biosynthetic enzymes in the pathway such as *PvANR1* and *PvLAR* and this determines PHD in *P*. *vulgaris*. The SNP in the mutant *Phvul.010G130600* gene found in ‘Wit-rood boontje’ causes a translational frameshift so that the C-terminus in the MYB it encodes lacks the EDLL motif and cannot interact with the coactivator complex to activate transcription of *PvANR1* and *PvLAR* which ultimately leads to a reduction or cessation of proanthocyanidin synthesis when it is in a homozygous condition.

This proposal is consistent with the higher levels of proanthocyanidins that were detected in seed coats of darkening pinto and cranberry beans compared to the slow darkening and non-darkening beans (Beninger et al. 2005; Chen et al. 2015; Freixas Coutin et al. 2017). Proanthocyanidins are abundant in the brown seed coats of many plants such as *A. thaliana* (Dixon et al. 2005) and *ANR* plays a key role in the biosynthesis of PAs in that species (Wang et al. 2018). *PvANR1* was found to be strongly associated with seed coat PA accumulation in darkening cranberry beans (Freixas Coutin et al. 2017). Loss of function of *ANR* gave large reductions in both soluble and insoluble PAs in *M. truncatula* seed coat (Liu et al. 2016). *LAR* along with *ANR* was found to contribute to PA polymerization in pea seed coats (Ferraro et al. 2014). However, the role of *LAR* in PA biosynthesis in *Phaseolus* remains unanswered.

The seed coat develops from the two integuments or outer layers of cells surrounding the ovule, which are derived from maternal tissue. The inner integument forms the segment, and the outer forms the testa. This means that, segregation for seed coat color always occurs one generation after seed formation (Erfatpour et al. 2018). As shown in Figure S4, the F_1_ seed with a seed coat genotype that is *jj* at the ND locus, has the same seed coat color as the female parent ‘Wit-rood boontje.’ For the F_2_ seed the seed coat tissue genotype is *Jj*. Therefore, F_2_ seeds have the darkening (RD) seed coat phenotype, but the embryo genotypes occur in the expected 1:2:1 ratio for *jj*/*Jj*/*JJ* individuals. Segregation for PHD can be observed in the F_3_. The dominant gene-based marker that was developed in the current study would enable bean breeders to differentiate F_2_ seeds or seedlings carrying the dominant allele (*JJ* and *Jj*) from those that are homozygous recessive (*jj*) genotypes in early generations (including F_2_ populations). In addition, this determination could be done at any stage of plant development, thus allowing selection at a seedling stage, rather than waiting to screen the phenotypic segregation in the UV treated F_3_ seed (Figure S4).

## Electronic supplementary material

Below is the link to the electronic supplementary material.

**Figure S1** Quality control for Illumina output using FASTQC software. (A) Per base sequence quality. The graph indicates that the sequence generated a high-quality score (>Q30) at each read position and the quality of the base calling remains stable along the read. (B) Per sequence quality scores. The average quality per read scored over 36 (PDF 73 kb)

**Figure S1** Quality control for Illumina output using FASTQC software. (A) Per base sequence quality. The graph indicates that the sequence generated a high-quality score (>Q30) at each read position and the quality of the base calling remains stable along the read. (B) Per sequence quality scores. The average quality per read scored over 36 (PDF 52 kb)

**Figure S2** Sequence alignment of a genomic segment shows a single nucleotide deletion (bordered by red lines) in a exon region (yellow shaded) in the gene *Phvul.010G133800* of three genotypes: ‘Wit-rood boontje’ (parent A with the non-darkening (ND) seed coat phenotype), RIL29 (ND), and Etna (regular darkening (RD) cranberry variety) in which a cytosine is present in 1533-15 (Parent B with the slow darkening (SD) seed coat phenotype), RIL81 (RD), Othello (RD pinto bean variety), and *P*. *vulgaris* reference genome (G19833). The single nucleotide polymorphism has limited capability for differentiating the RD beans from the ND beans because the RD Etna shows the same pattern as the ND genotypes (PDF 168 kb)

**Figure S2** Sequence alignment of a genomic segment shows a single nucleotide deletion (bordered by red lines) in a exon region (yellow shaded) in the gene *Phvul.010G133800* of three genotypes: ‘Wit-rood boontje’ (parent A with the non-darkening (ND) seed coat phenotype), RIL29 (ND), and Etna (regular darkening (RD) cranberry variety) in which a cytosine is present in 1533-15 (Parent B with the slow darkening (SD) seed coat phenotype), RIL81 (RD), Othello (RD pinto bean variety), and *P*. *vulgaris* reference genome (G19833). The single nucleotide polymorphism has limited capability for differentiating the RD beans from the ND beans because the RD Etna shows the same pattern as the ND genotypes (PDF 119 kb)

**Figure S3** Schematic representation of a MBW (MYB-bHLH-WD40) complex involved in regulation of flavonoid biosynthesis pathway genes (modified from REF). (A) The N-terminal MYB-interacting region (MIR) of the bHLH binds to the bHLH-binding motif in the MYB R3 repeat and forms a ternary complex with a WDR. (A1) R2R3-MYB proteins have a conserved DNA-binding domain (DBD) at the N-terminus and a highly variable, C-terminal transcriptional activation domain (TAD). (A2) Basic helix-loop-helix (bHLH) proteins are characterized by MIR and WD40-binding motif (comprising the TAD) at the N-terminal end and a bHLH domain at the C-terminus. (A3) a WD40 protein with a WD40 repeat. (B) A R2R3-MYB protein can bind directly to an upstream enhancer sequence as an activator, either on its own or in the form of a MBW complex. A R2R3-MYB protein bound to DNA can interact with other proteins (coactivators) via its transcriptional activation domain to activate RNA polymerase II (red) and thus transcription. The DNA can loop around on itself to cause this interaction between a R2R3-MYB protein and coactivators that mediate the activity of RNA polymerase (TIFF 387 kb)

**Figure S4** Seed coat phenotypes for F_1_, F_2_, and F_3_ seeds derived from a ‘Wit-rood boontje × 1533-15 cross. Segregation for the seed coat darkening trait is observed in the F_3_ which contained three distinct seed coat phenotypes [regular darkening (RD), slow darkening (SD), and non-darkening (ND)] (TIFF 2958 kb)

Supplementary material 7 (DOCX 22 kb)
