# Supplementary figures and images for: A R2R3-MYB gene-based marker for the non-darkening seed coat trait in pinto and cranberry beans (Phaseolus vulgaris L.) derived from ‘Wit-rood boontje’
Source: Theor Appl Genet. 2020 Feb 28;133(6):1977–94. doi: 10.1007/s00122-020-03571-7 (PMC7237406; doi:10.1007/s00122-020-03571-7)

## Quality scores across all bases (Illumina 1.9 encoding)

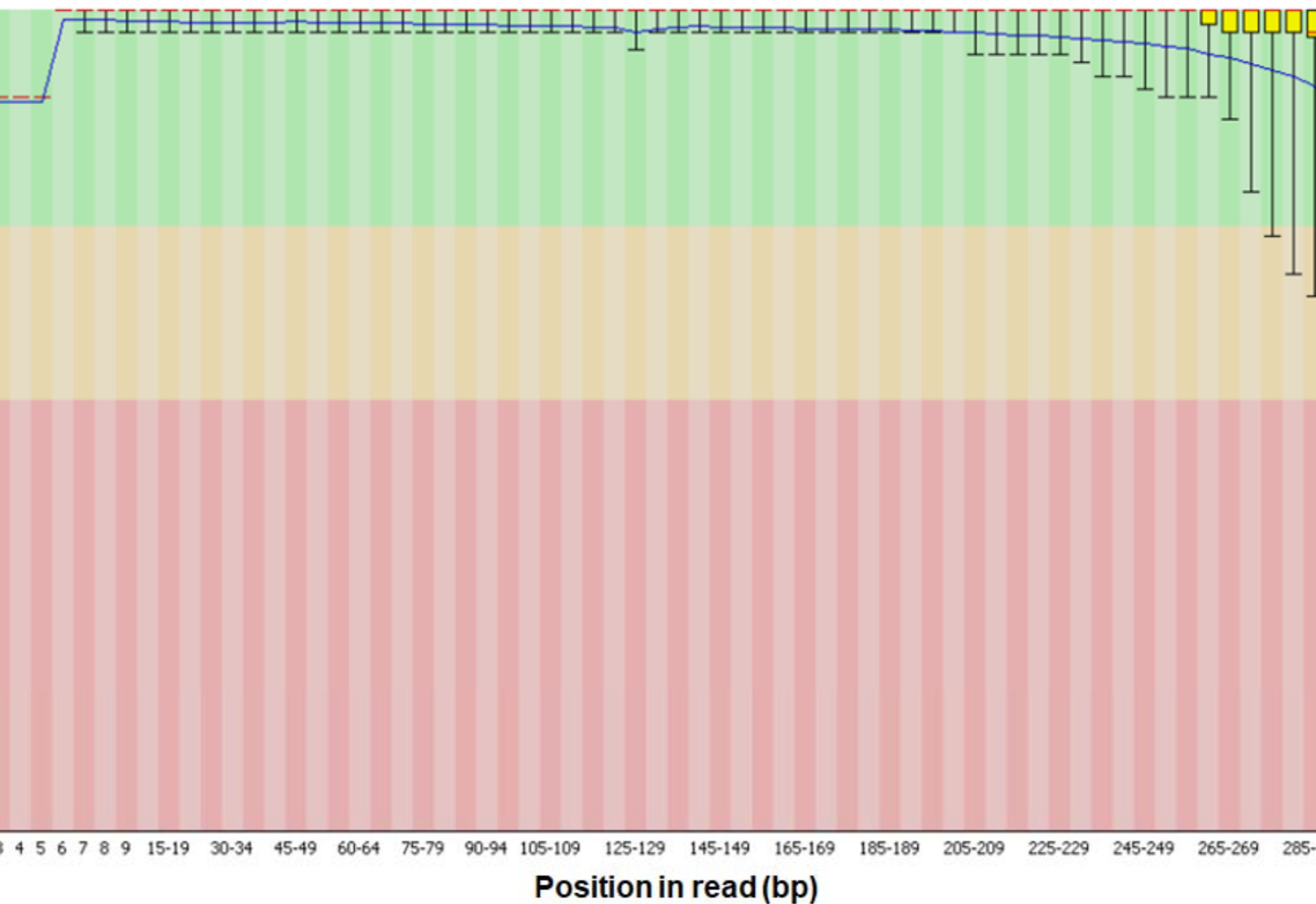

Supplement: Supplementary file 1 — Figure S1 Quality control for Illumina output using FASTQC software. (A) Per base sequence quality. The graph indicates that the sequence generated a high-quality score (>Q30) at each read position and the quality of the base calling remains stable along the read. (B) Per sequence quality scores. The average quality per read scored over 36 (PDF 73 kb) [file 122_2020_3571_MOESM1_ESM.pdf]

**B**

## Quality scores distribution over all sequences

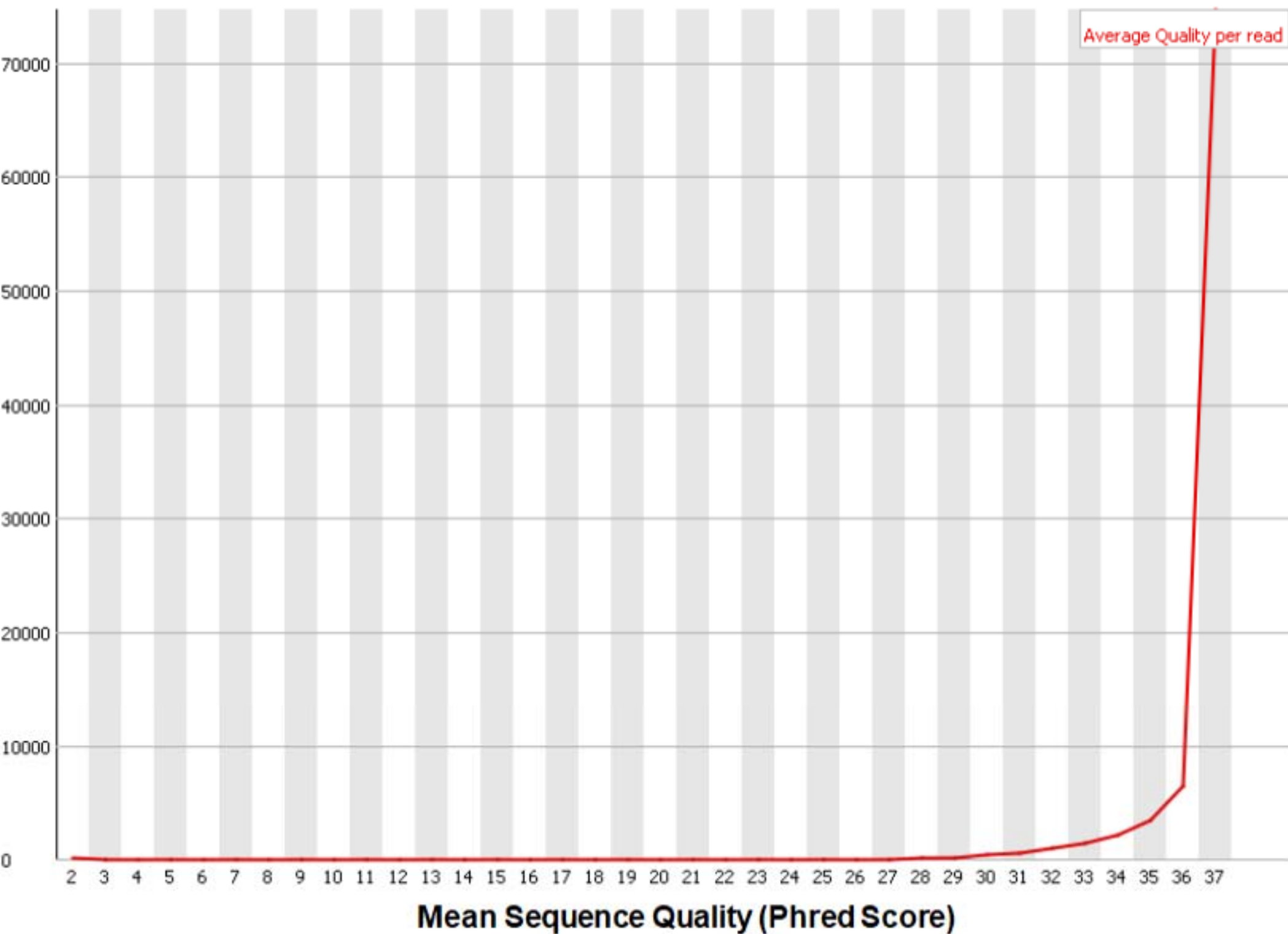

Supplement: Supplementary file 2 — Figure S1 Quality control for Illumina output using FASTQC software. (A) Per base sequence quality. The graph indicates that the sequence generated a high-quality score (>Q30) at each read position and the quality of the base calling remains stable along the read. (B) Per sequence quality scores. The average quality per read scored over 36 (PDF 52 kb) [file 122_2020_3571_MOESM2_ESM.pdf]

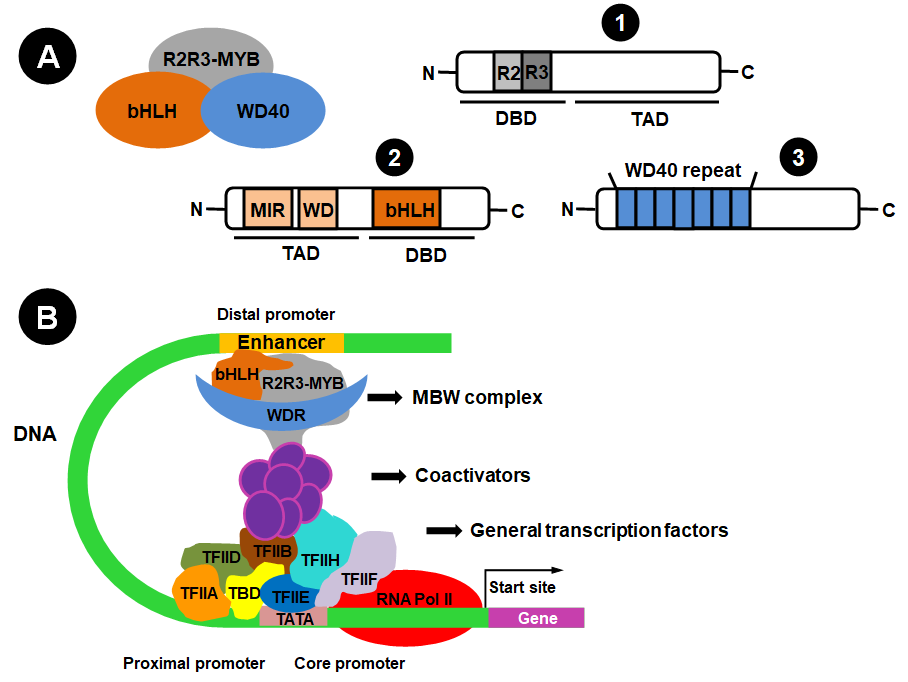

Supplement: Supplementary file 5 — Figure S3 Schematic representation of a MBW (MYB-bHLH-WD40) complex involved in regulation of flavonoid biosynthesis pathway genes (modified from REF). (A) The N-terminal MYB-interacting region (MIR) of the bHLH binds to the bHLH-binding motif in the MYB R3 repeat and forms a ternary complex with a WDR. (A1) R2R3-MYB proteins have a conserved DNA-binding domain (DBD) at the N-terminus and a highly variable, C-terminal transcriptional activation domain (TAD). (A2) Basic helix-loop-helix (bHLH) proteins are characterized by MIR and WD40-binding motif (comprising the TAD) at the N-terminal end and a bHLH domain at the C-terminus. (A3) a WD40 protein with a WD40 repeat. (B) A R2R3-MYB protein can bind directly to an upstream enhancer sequence as an activator, either on its own or in the form of a MBW complex. A R2R3-MYB protein bound to DNA can interact with other proteins (coactivators) via its transcriptional activation domain to activate RNA polymerase II (red) and thus transcription. The DNA can loop around on itself to cause this interaction between a R2R3-MYB protein and coactivators that mediate the activity of RNA polymerase (TIFF 387 kb) [file 122_2020_3571_MOESM5_ESM.tif]

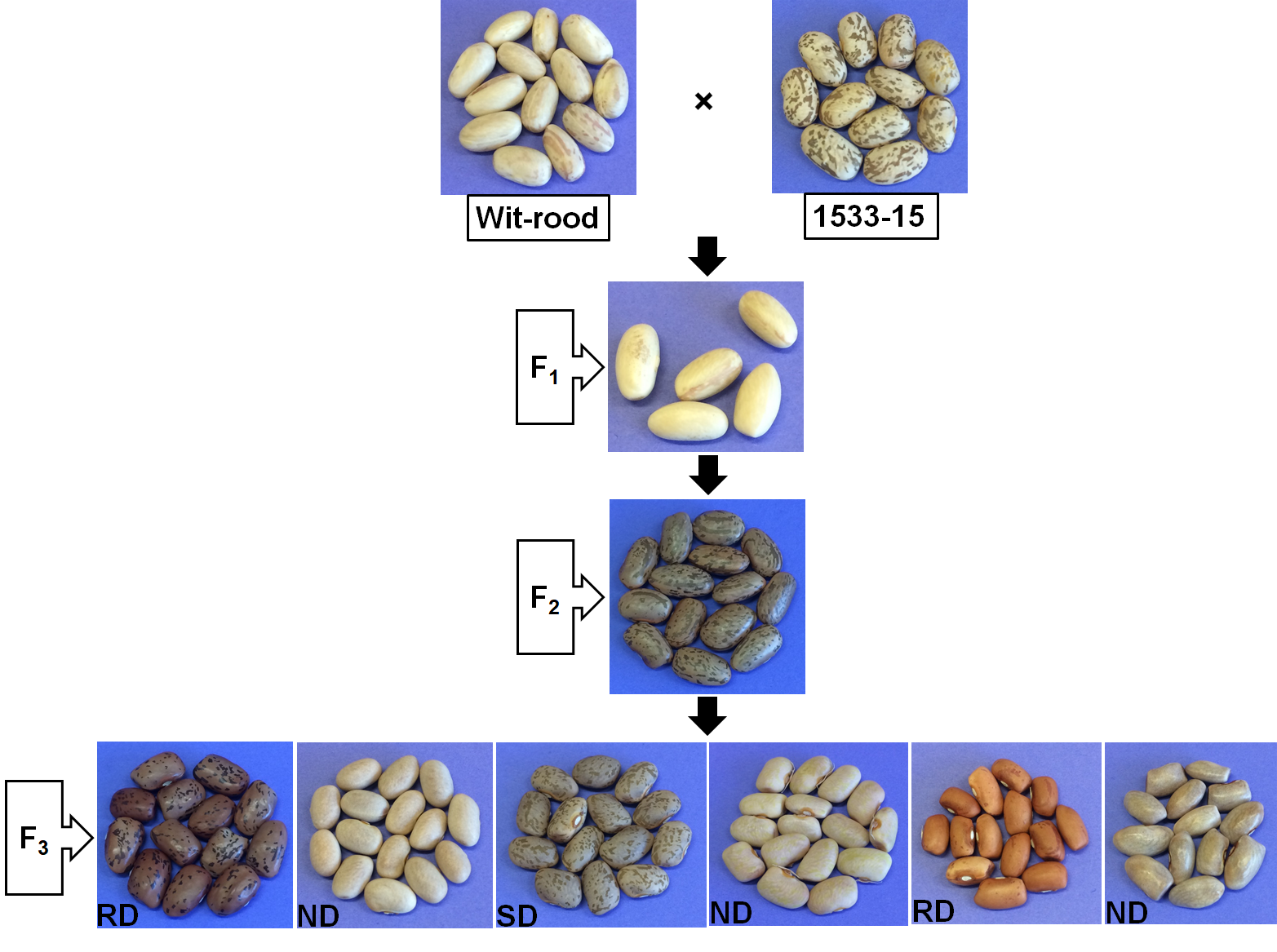

Supplement: Supplementary file 6 — Figure S4 Seed coat phenotypes for F1, F2, and F3 seeds derived from a ‘Wit-rood boontje × 1533-15 cross. Segregation for the seed coat darkening trait is observed in the F3 which contained three distinct seed coat phenotypes [regular darkening (RD), slow darkening (SD), and non-darkening (ND)] (TIFF 2958 kb) [file 122_2020_3571_MOESM6_ESM.tif]
