# Supplementary material for: A R2R3-MYB gene-based marker for the non-darkening seed coat trait in pinto and cranberry beans (Phaseolus vulgaris L.) derived from ‘Wit-rood boontje’
Source: Theor Appl Genet. 2020 Feb 28;133(6):1977–94. doi: 10.1007/s00122-020-03571-7 (PMC7237406; doi:10.1007/s00122-020-03571-7)
Supplement: Supplementary file 3 — Figure S2 Sequence alignment of a genomic segment shows a single nucleotide deletion (bordered by red lines) in a exon region (yellow shaded) in the gene Phvul.010G133800 of three genotypes: ‘Wit-rood boontje’ (parent A with the non-darkening (ND) seed coat phenotype), RIL29 (ND), and Etna (regular darkening (RD) cranberry variety) in which a cytosine is present in 1533-15 (Parent B with the slow darkening (SD) seed coat phenotype), RIL81 (RD), Othello (RD pinto bean variety), and P. vulgaris reference genome (G19833). The single nucleotide polymorphism has limited capability for differentiating the RD beans from the ND beans because the RD Etna shows the same pattern as the ND genotypes (PDF 168 kb) [file 122_2020_3571_MOESM3_ESM.pdf]

```
RIL29 ND                 tgagattttggtttcacgttattaaaaaaagaaatatgataatccatatttgaatacat 420
1533-15 SD               tgagattttggtttcacgttattaaaaaaagaaatatgataatccatatttgaatacat 420
RIL81 RD                 tgagattttggtttcacgttattaaaaaaagaaatatgataatccatatttgaatacat 420
Othello RD               tgagattttggtttcacgttattaaaaaaagaaatatgataatccatatttgaatacat 420
Etna RD                  tgagattttggtttcacgttattaaaaaaagaaatatgataatccatatttgaatacat 420
G19833                   tgagattttggtttcacgttattaaaaaaagaaatatgataatccatatttgaatacat 420
                         ************************************************************

Wit-rood boontje ND      gcatgcatgcaacttaaagttctaaatagtttttataatttggtgaagGCATGGAGATC 480
RIL29 ND                 gcatgcatgcaacttaaagttctaaatagtttttataatttggtgaagGCATGGAGATC 480
1533-15 SD               gcatgcatgcaacttaaagttctaaatagtttttataatttggtgaagGCATGGAGATC 480
RIL81 RD                 gcatgcatgcaacttaaagttctaaatagtttttataatttggtgaagGCATGGAGATC 480
Othello RD               gcatgcatgcaacttaaagttctaaatagtttttataatttggtgaagGCATGGAGATC 480
Etna RD                  gcatgcatgcaacttaaagttctaaatagtttttataatttggtgaagGCATGGAGATC 480
G19833                   gcatgcatgcaacttaaagttctaaatagtttttataatttggtgaagGCATGGAGATC 480
                         ************************************************************

Wit-rood boontje ND      CATCAATTACGGTGAACGTTGCAGGCGAGGAGAAAGAGGAGTCAAAAGGGTGGAGAAAAG 540
RIL29 ND                 CATCAATTACGGTGAACGTTGCAGGCGAGGAGAAAGAGGAGTCAAAAGGGTGGAGAAAAG 540
1533-15 SD               CATCAATTACGGTGAACGTTGCAGGCGAGGAGAAAGAGGAGTCAAAAGGGTGGAGAAAAG 540
RIL81 RD                 CATCAATTACGGTGAACGTTGCAGGCGAGGAGAAAGAGGAGTCAAAAGGGTGGAGAAAAG 540
Othello RD               CATCAATTACGGTGAACGTTGCAGGCGAGGAGAAAGAGGAGTCAAAAGGGTGGAGAAAAG 540
Etna RD                  CATCAATTACGGTGAACGTTGCAGGCGAGGAGAAAGAGGAGTCAAAAGGGTGGAGAAAAG 540
G19833                   CATCAATTACGGTGAACGTTGCAGGCGAGGAGAAAGAGGAGTCAAAAGGGTGGAGAAAAG 540
                         ************************************************************

Wit-rood boontje ND      CTCTGAAAAAATGTAGGGAATTGGCTGGCACACAAGGATAAGGATAAATGGTTGAAGGATA 600
RIL29 ND                 CTCTGAAAAAATGTAGGGAATTGGCTGGCACACAAGGATAAGGATAAATGGTTGAAGGATA 600
1533-15 SD               CTCTGAAAAAATGTAGGGAATTGGCTGGCACACAAGGATAAGGATAAATGGTTGAAGGATA 600
RIL81 RD                 CTCTGAAAAAATGTAGGGAATTGGCTGGCACACAAGGATAAGGATAAATGGTTGAAGGATA 600
Othello RD               CTCTGAAAAAATGTAGGGAATTGGCTGGCACACAAGGATAAGGATAAATGGTTGAAGGATA 600
Etna RD                  CTCTGAAAAAATGTAGGGAATTGGCTGGCACACAAGGATAAGGATAAATGGTTGAAGGATA 600
G19833                   CTCTGAAAAAATGTAGGGAATTGGCTGGCACACAAGGATAAGGATAAATGGTTGAAGGATA 600
                         ************************************************************

Wit-rood boontje ND      TGAGAGGTAACCTTTCTTTGGTGGCTACTGTAATCACAACAATGACGTTTCAAAATGCTC 660
RIL29 ND                 TGAGAGGTAACCTTTCTTTGGTGGCTACTGTAATCACAACAATGACGTTTCAAAATGCTC 660
1533-15 SD               TGAGAGGTAACCTTTCTTTGGTGGCTACTGTAATCACAACAATGACGTTTCAAAATGCTC 660
RIL81 RD                 TGAGAGGTAACCTTTCTTTGGTGGCTACTGTAATCACAACAATGACGTTTCAAAATGCTC 660
Othello RD               TGAGAGGTAACCTTTCTTTGGTGGCTACTGTAATCACAACAATGACGTTTCAAAATGCTC 660
Etna RD                  TGAGAGGTAACCTTTCTTTGGTGGCTACTGTAATCACAACAATGACGTTTCAAAATGCTC 660
G19833                   TGAGAGGTAACCTTTCTTTGGTGGCTACTGTAATCACAACAATGACGTTTCAAAATGCTC 660
                         ************************************************************

Wit-rood boontje ND      TAAACCCACCTGGTGGTGTTAGACCGCCAAGAGAGAGTGAAGGAAAAGTGGTTTGTTCAG 720
RIL29 ND                 TAAACCCACCTGGTGGTGTTAGACCGCCAAGAGAGAGTGAAGGAAAAGTGGTTTGTTCAG 720
1533-15 SD               TAAACCCACCTGGTGGTGTTAGACCGCCAAGAGAGAGTGAAGGAAAAGTGGTTTGTTCAG 720
RIL81 RD                 TAAACCCACCTGGTGGTGTTAGACCGCCAAGAGAGAGTGAAGGAAAAGTGGTTTGTTCAG 720
Othello RD               TAAACCCACCTGGTGGTGTTAGACCGCCAAGAGAGAGTGAAGGAAAAGTGGTTTGTTCAG 720
Etna RD                  TAAACCCACCTGGTGGTGTTAGACCGCCAAGAGAGAGTGAAGGAAAAGTGGTTTGTTCAG 720
G19833                   TAAACCCACCTGGTGGTGTTAGACCGCCAAGAGAGAGTGAAGGAAAAGTGGTTTGTTCAG 720
                         ************************************************************

Wit-rood boontje ND      ACGATATCTGGCCATGTCCTGGAGAATCTATCCTAGCTTATAGAATGCCAGGGCATTATA 780
RIL29 ND                 ACGATATCTGGCCATGTCCTGGAGAATCTATCCTAGCTTATAGAATGCCAGGGCATTATA 780
1533-15 SD               ACGATATCTGGCCATGTCCTGGAGAATCTATCCTAGCTTATAGAATGCCAGGGCATTATA 780
RIL81 RD                 ACGATATCTGGCCATGTCCTGGAGAATCTATCCTAGCTTATAGAATGCCAGGGCATTATA 780
Othello RD               ACGATATCTGGCCATGTCCTGGAGAATCTATCCTAGCTTATAGAATGCCAGGGCATTATA 780
Etna RD                  ACGATATCTGGCCATGTCCTGGAGAATCTATCCTAGCTTATAGAATGCCAGGGCATTATA 780
G19833                   ACGATATCTGGCCATGTCCTGGAGAATCTATCCTAGCTTATAGAATGCCAGGGCATTATA 780
                         ************************************************************
```
