# Supplementary material for: A R2R3-MYB gene-based marker for the non-darkening seed coat trait in pinto and cranberry beans (Phaseolus vulgaris L.) derived from ‘Wit-rood boontje’
Source: Theor Appl Genet. 2020 Feb 28;133(6):1977–94. doi: 10.1007/s00122-020-03571-7 (PMC7237406; doi:10.1007/s00122-020-03571-7)
Supplement: Supplementary file 4 — Figure S2 Sequence alignment of a genomic segment shows a single nucleotide deletion (bordered by red lines) in a exon region (yellow shaded) in the gene Phvul.010G133800 of three genotypes: ‘Wit-rood boontje’ (parent A with the non-darkening (ND) seed coat phenotype), RIL29 (ND), and Etna (regular darkening (RD) cranberry variety) in which a cytosine is present in 1533-15 (Parent B with the slow darkening (SD) seed coat phenotype), RIL81 (RD), Othello (RD pinto bean variety), and P. vulgaris reference genome (G19833). The single nucleotide polymorphism has limited capability for differentiating the RD beans from the ND beans because the RD Etna shows the same pattern as the ND genotypes (PDF 119 kb) [file 122_2020_3571_MOESM4_ESM.pdf]

```
Othello RD             CCTTCTTCCTTATTTGGAATACTACATGTTTCATATCATCTTCAGTTGTTTGTCTCTTGC 840
Etna RD                CCTTCTTCCTTATTTGGAATACTACATGTTTCATATCATCTTCAGTTGTTTGTCTCTTGC 840
G19833                 CCTTCTTCCTTATTTGGAATACTACATGTTTCATATCATCTTCAGTTGTTTGTCTCTTGC 840
                       ************************************************************

Wit-rood boontje ND    TTGTTAGTGGATTCCCTCTCAACCACAGATTTTTCACCTGGCTTTTATCAATAGGTATGT 900
RIL29 ND               TTGTTAGTGGATTCCCTCTCAACCACAGATTTTTCACCTGGCTTTTATCAATAGGTATGT 900
1533-15 SD             TTGTTAGTGGATTCCCTCTCAACCACAGATTTTTCACCTGGCTTTTATCAATAGGTATGT 900
RIL81 RD               TTGTTAGTGGATTCCCTCTCAACCACAGATTTTTCACCTGGCTTTTATCAATAGGTATGT 900
Othello RD             TTGTTAGTGGATTCCCTCTCAACCACAGATTTTTCACCTGGCTTTTATCAATAGGTATGT 900
Etna RD                TTGTTAGTGGATTCCCTCTCAACCACAGATTTTTCACCTGGCTTTTATCAATAGGTATGT 900
G19833                 TTGTTAGTGGATTCCCTCTCAACCACAGATTTTTCACCTGGCTTTTATCAATAGGTATGT 900
                       ************************************************************

Wit-rood boontje ND    GCATTACCATCACTAGTCTTGCTCTTACCTACATGTATGGTGTTAAAATGGTCACCCCTG 960
RIL29 ND               GCATTACCATCACTAGTCTTGCTCTTACCTACATGTATGGTGTTAAAATGGTCACCCCTG 960
1533-15 SD             GCATTACCATCACTAGTCTTGCTCTTACCTACATGTATGGTGTTAAAATGGTCACCCCTG 960
RIL81 RD               GCATTACCATCACTAGTCTTGCTCTTACCTACATGTATGGTGTTAAAATGGTCACCCCTG 960
Othello RD             GCATTACCATCACTAGTCTTGCTCTTACCTACATGTATGGTGTTAAAATGGTCACCCCTG 960
Etna RD                GCATTACCATCACTAGTCTTGCTCTTACCTACATGTATGGTGTTAAAATGGTCACCCCTG 960
G19833                 GCATTACCATCACTAGTCTTGCTCTTACCTACATGTATGGTGTTAAAATGGTCACCCCTG 960
                       ************************************************************

Wit-rood boontje ND    ATCCTGTCTGGGCCACATCCATCTCCATGTTTGGAATTGTTATTATTATTATTTGGA-TG 1019
RIL29 ND               ATCCTGTCTGGGCCACATCCATCTCCATGTTTGGAATTGTTATTATTATTATTTGGA-TG 1019
1533-15 SD             ATCCTGTCTGGGCCACATCCATCTCCATGTTTGGAATTGTTATTATTATTATTTGGATTG 1019
RIL81 RD               ATCCTGTCTGGGCCACATCCATCTCCATGTTTGGAATTGTTATTATTATTATTTGGATGG 1020
Othello RD             ATCCTGTCTGGGCCACATCCATCTCCATGTTTGGAATTGTTATTATTATTATTTGGATGG 1020
Etna RD                ATCCTGTCTGGGCCACATCCATCTCCATGTTTGGAATTGTTATTATTATTATTTGGA-GG 1020
G19833                 ATCCTGTCTGGGCCACATCCATCTCCATGTTTGGAATTGTTATTATTATTATTTGGATGG 1020
                       *********************************************************  *

Wit-rood boontje ND    CACTGCTAGTACTCGTGACTTTTGTTTTTTGCTTGCGCCTGGTTATTTGGATTCTCACTA 1079
RIL29 ND               CACTGCTAGTACTCGTGACTTTTGTTTTTTGCTTGCGCCTGGTTATTTGGATTCTCACTA 1079
1533-15 SD             CACTGCTAGTACTCGTGACTTTTGTTTTTTGCTTGCGCCTGGTTATTTGGATTCTCACTA 1079
RIL81 RD               CACTGCTAGTACTCGTGACTTTTGTTTTTTGCTTGCGCCTGGTTATTTGGATTCTCACTA 1080
Othello RD             [illegible] 1080
```
