# Supplementary material for: A R2R3-MYB gene-based marker for the non-darkening seed coat trait in pinto and cranberry beans (Phaseolus vulgaris L.) derived from ‘Wit-rood boontje’
Source: Theor Appl Genet. 2020 Feb 28;133(6):1977–94. doi: 10.1007/s00122-020-03571-7 (PMC7237406; doi:10.1007/s00122-020-03571-7)
Supplement: Supplementary file 7 — Supplementary material 7 (DOCX 22 kb) [file 122_2020_3571_MOESM7_ESM.docx]

**Table S1** Primer sets used for amplicon generation from each candidate gene, their sequence length (bp), melting temperature (Tm), and GC content.

| Primer/fragment size | Sequence (5ʹ-3ʹ) | Length (bp) | Tm (ºC) | GC% |
| --- | --- | --- | --- | --- |
| P1- Phvul.01G130300 | F: TCATCATTGTCACAATCTCTTGGC | 21 | 62 | 47.6 |
| (1175 bp) | R: GAGAAGAGACCTTGGATTTGTGTG | 23 | 62 | 47.8 |
|  |  |  |  |  |
| P2- Phvul.010G130300 | F: CTGTTCTTATTCCCTCTACCATCTAC | 26 | 62 | 42.3 |
| (1205 bp) | R: GTGATTATATGCATGTCCCAGTTTC | 25 | 62 | 40 |
|  |  |  |  |  |
| P1- Phvul.010G130500 | F: GACTACTTACTTCCACGTGCTC | 22 | 62 | 50 |
| (1314 bp) | R: ATTGGTTCATAGCCAATCAAATCC | 24 | 62 | 37.5 |
|  |  |  |  |  |
| P1- Phvul.010G130600 | F: GGTAGTGTGTGTAGTGAGAAGAAG | 24 | 62 | 45.8 |
| (1453 bp) | R: AGAGAAGAGACCTTGGATTTGTG | 23 | 62 | 43.5 |
|  |  |  |  |  |
| P1- Phvul.010G130700 | F: AGATTGTGTGTGTTTGTGTCTTG | 23 | 62 | 39.1 |
| (1404 bp) | R: TAAACCCGTAAGGCAACTCAG | 21 | 62 | 47.6 |
|  |  |  |  |  |
| P2- Phvul.010G130700 | F: CGCATCACTGTCGTCCAAAC | 20 | 63 | 55 |
| (1400 bp) | R: GCCACACTATAGCTTGGGAAAG | 22 | 63 | 50 |
|  |  |  |  |  |
| P3- Phvul.010G130700 | F: GTCACGACACTGGTATAATTGC | 22 | 61 | 45.5 |
| (1554 bp) | R: GTGAACTCTGCTGTGACATTTG | 22 | 62 | 45.5 |
|  |  |  |  |  |
| P4- Phvul.010G130700 | F: AATGAGTCAGGGTCAAATCAGG | 22 | 62 | 45.5 |
| (1546 bp) | R: CTGGTTTCCTAAAGTGGAGGAC | 22 | 62 | 50 |
|  |  |  |  |  |
| P5- Phvul.010G130700 | F: TTGGTACCCTAGGCAATGAAG | 21 | 62 | 47.6 |
| (1562 bp) | R: GCCACTGTTGCTGTGTTAAG | 20 | 62 | 50 |
|  |  |  |  |  |
| P6- Phvul.010G130700 | F: GGTTTACCAAGGTCATCATCCC | 22 | 63 | 50 |
| (1516 bp) | R: TGCAACCGTGTGTAGTTGTC | 22 | 63 | 50 |
|  |  |  |  |  |
| P7- Phvul.010G130700 | F: GCAGACAACTACACACGGTTG | 21 | 63 | 52.4 |
| (1406 bp) | R: AGGCATCTTACCAGGTATACGG | 22 | 63 | 50 |
|  |  |  |  |  |
| P8- Phvul.010G130700 | F: AGCTGGTTCTCAAATGCTACAG | 22 | 63 | 45.5 |
| (1282 bp) | R: ATGGTTGCTTTACACTCGATCC | 22 | 63 | 45.5 |
|  |  |  |  |  |
| P1- Phvul.010G131300 | F: CAATCTTCTCTTCTCAGACCTACTC | 25 | 62 | 44 |
| (1715 bp) | R: ACTTCAAATCCTTCTCCACCTC | 22 | 62 | 45.5 |
|  |  |  |  |  |
| P2- Phvul.010G131300 | F: GTGGAGGTGGAGAAGGATTTG | 21 | 62 | 52.4 |
| (1310 bp) | R: TAGCAACAGCAATAACCTGAGAG | 23 | 62 | 43.5 |
|  |  |  |  |  |
| P3- Phvul.010G131300 | F: GTTCTCTCAGGTTATTGCTGTTG | 23 | 61 | 43.5 |
| (1414 bp) | R: TGGGTTGACGAGCTAATCTG | 20 | 62 | 50 |
|  |  |  |  |  |
| P4- Phvul.010G131300 | F: GGACAGATTAGCTCGTCAACC | 21 | 62 | 52.4 |
| (1100 bp) | R: GCAATGCCCGTTTCCTTTAC | 20 | 62 | 50 |
|  |  |  |  |  |
| P1- Phvul.010G131400 | F: ATACCACTCTCCACTCTCTCTC | 22 | 62 | 50 |
| (1532 bp) | R: CTCCCACCTCCATTACTTCATC | 22 | 62 | 50 |
|  |  |  |  |  |
| P2- Phvul.010G131400 | F: TGAAGGTGATGGAAGGTTAGTG | 22 | 62 | 45.5 |
| (1418 bp) | R: GGACAAACCTAAGTCATTTGTTAGC | 25 | 62 | 40 |
|  |  |  |  |  |
| P3- Phvul.010G131400 | F: TGTGGTAATGCAGTAAGATGTAGC | 24 | 63 | 41.7 |
| (1422 bp) | R: CCATATCTTTCCTTGGACGACTTC | 24 | 63 | 45.8 |
|  |  |  |  |  |
| P4- Phvul.010G131400 | F: TGGAGGTTCTGATGTCCAAATTC | 23 | 63 | 43.5 |
| (1201 bp) | R: GCTCAATTGTGCCTACTACTGG | 22 | 63 | 50 |
|  |  |  |  |  |
| P5- Phvul.010G131400 | F: GAGAAGCAGTGTCCAGTAGTAG | 22 | 61 | 50 |
| (1124 bp) | R: AACCTGACATCATTCTATTCAACG | 24 | 61 | 37.5 |
|  |  |  |  |  |
| P1- Phvul.010G133100 | F: AGGGTATACCAGCATACCACAATTC | 25 | 64 | 44 |
| (712 bp) | R: TGTCTTGAGTGGTGCCTTCTTC | 22 | 64 | 50 |
|  |  |  |  |  |
| P1- Phvul.010G132300 | F: AGTTCTATGCATTTGCTTAAAGGAC | 25 | 62 | 36 |
| (720 bp) | R: TGAACGGTAGCCATGAATGAG | 21 | 62 | 47.6 |
